# Fast–Slow Bursters in the Unfolding of a High Codimension Singularity and the Ultra-slow Transitions of Classes

**DOI:** 10.1186/s13408-017-0050-8

**Published:** 2017-07-25

**Authors:** Maria Luisa Saggio, Andreas Spiegler, Christophe Bernard, Viktor K. Jirsa

**Affiliations:** 0000 0001 2176 4817 grid.5399.6 INS, Institut de Neurosciences des Systèmes, Inserm, Aix Marseille Univ, Marseille, France

**Keywords:** Bursting, Time-scale separation, Degenerate and doubly degenerate Takens–Bogdanov singularity, Ultra-slow modulation, Unfolding theory, Minimal models

## Abstract

Bursting is a phenomenon found in a variety of physical and biological systems. For example, in neuroscience, bursting is believed to play a key role in the way information is transferred in the nervous system. In this work, we propose a model that, appropriately tuned, can display several types of bursting behaviors. The model contains two subsystems acting at different time scales. For the fast subsystem we use the planar unfolding of a high codimension singularity. In its bifurcation diagram, we locate paths that underlie the right sequence of bifurcations necessary for bursting. The slow subsystem steers the fast one back and forth along these paths leading to bursting behavior. The model is able to produce almost all the classes of bursting predicted for systems with a planar fast subsystem. Transitions between classes can be obtained through an ultra-slow modulation of the model’s parameters. A detailed exploration of the parameter space allows predicting possible transitions. This provides a single framework to understand the coexistence of diverse bursting patterns in physical and biological systems or in models.

## Introduction

**Fig. 1:**
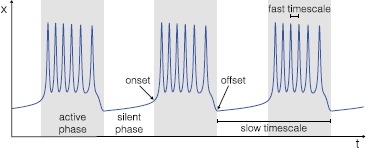
Bursting activity. Bursters are characterized by the alternation between active (*gray boxes*) and silent (*white*) phases. In fast–slow bursters we can distinguish two rhythms: one within the active phase, the fast time scale, and the other between active and silent phases, the slow time scale. Oscillations start at the onset of the active phase and terminate at its offset

Many systems in nature can display bursts of activity that alternate with silent behavior [1, 2]. An example of bursting is shown in Fig. 1. Bursting is in fact part of the dynamical repertoire of many chemical and biological systems and is the primary mode of electrical activity in several neurons and endocrine cells [3–11]. Neuronal bursting, in particular, is of key importance for the production of motor, sensory and cognitive behavior [12]. Bursts of activity are central to information processing, as they produce reliable synaptic transmission and as they can facilitate synaptic plasticity [13]. Bursting can also be pathological. For example, epileptiform discharges are associated with bursts of neural ensembles with highly synchronized activity [14].

Modeling bursting behavior can help to uncover the mechanisms underlying the bursting dynamics in complex systems. Moreover, modeling gives the opportunity to perform in silico experiments to predict the outcome of manipulations of the system. For example, the Epileptor [15], which is a phenomenological model for the most common bursting behavior in epilepsy, has been used to predict seizure propagation and recruitment in highly personalized virtual epileptic brains [16]. Different treatment strategies can be tested in silico in these virtual epileptic patients, such as interventions on the network topology, stimulations and parameter changes, providing a tool throughout the presurgical evaluation.

Bursting activities, though, can present large differences, such as differences in amplitude and frequency. Different properties at the onset and offset of the burst (i.e. active phase; see the gray boxes in Fig. 1) have been linked to specific qualitative changes in the dynamics, which correspond to bifurcations occurring in a subsystem of the dynamical system [1, 17]. Izhikevich used the onset/offset bifurcations pair criterion to compile a taxonomy of possible bursting classes [17]. In the present study we provide a single autonomous model, comprising a minimal number of variables and parameters, able to produce many classes from this taxonomy. For this purpose, we make use of (i) the ‘dissection’ method applied by Rinzel [18] to the study of fast–slow bursters, namely bursters for which there is a time-scale separation between the rhythm of oscillation within the active phase and the rhythm at which silent and active phases alternate; (ii) the unfolding theory approach proposed by Golubitsky et al. [19], based on the idea that the bifurcations involved in bursting activity can be ‘collapsed to a single local bifurcation, generally of higher codimension’.

Section 2 is a brief review of results in the literature upon which our work is built. In this section we will briefly recall both the dissection method [18] and the unfolding theory approach applied to bursting activity [19–21]. We will also describe a codimension 3 singularity, the degenerate Takens–Bogdanov (codim-3 deg. TB) singularity [22–24], for which we recapitulate the results of the application of the unfolding approach for bursting activity [3, 19, 20, 25]. In Sect. 3 we will systematically extend the unfolding approach to the deg. TB singularity and show how this allows for a rich repertoire of bursting classes. The model in fact is able to display almost all types of bursting behavior that have been predicted for systems with time-scale separation and a planar subsystem acting on the fast time scale [17]. We will explain in detail how to build the different classes of bursters. Furthermore, we will show how to obtain transitions among classes with an ultra-slow modulation of the model parameters, as done for a conceptually similar model by Franci et al. [21]. In addition, we will show additional bursting classes obtained when varying a fourth parameter of the model, which correspond to exploring the codimension 4 doubly degenerate TB singularity [20, 26]. Finally, we will apply a measure for complexity based on codimensions [19] to the bursting classes found in the model. This can help to understand the occurrence of bursting phenomena in empirical data and models.

## Modeling Fast–Slow Bursters

### Dissection Method

At least two rhythms characterize a burster: the fast rhythm of the oscillations within the active phase, and the slower rhythm of the alternation between active and silent phases.

If the time scales of these two rhythms are sufficiently apart, we have a *fast–slow* burster. Rinzel [27] took advantage of this separation to analyze bursting in the Chay–Keyzer model for pancreatic *β* cells. He applied a powerful method, called ‘dissection’, that is at the base of our work. The idea behind this method is that we can distinguish two subsystems depending on the time scale at which they act, the slow and the fast ones, and that the variables of the slow subsystem enter the fast subsystem’s equation as parameters.

The fast–slow burster can be described by

1 $$ \textstyle\begin{cases} \dot{\mathbf{x}}=f(\mathbf{x},\mathbf{z}),\\ \dot{\mathbf{z}}=cg(\mathbf{x},\mathbf{z}), \end{cases}\displaystyle \quad c\ll1, $$

where $\mathbf{x}=\mathbf{x}(t)\in\mathbb{R}^{n}$ is the state vector of fast variables and $\mathbf{z}=\mathbf{z}(t)\in\mathbb{R}^{m}$ is the vector of slow ones. The dot above a variable indicates the time derivative $d/dt$, *f* and *g* are functions, and $c=1/\tau$ is the inverse of the characteristic time constant *τ* of the separation between the two rhythms, that is, the ratio between the slow and fast time scales. To have time-scale separation, with **x** and **z** as fast and slow variables, respectively, we need to have $c\ll1$.

Both the fast and the slow subsystems can be analyzed in isolation. One can thus build a bifurcation diagram showing how the state space topology of the fast subsystem changes for different values of the slow variables **z**, here playing the role of bifurcation parameters. If the time-scale separation holds, the coupling with the slowly changing **z** moves the fast subsystem **x** in this bifurcation diagram, without affecting the topology of the latter.

### Classification of Bursters

When coupled together, the two subsystems must fulfill at least two requirements to produce bursting activity. First, the fast subsystem should be able to display both silent and oscillatory activity depending on the value of its parameters, that is, the slow variables [25]. This implies that the dimensionality of the fast subsystem should be $n\geqslant2$, to allow for the existence of a limit cycle. Second, the slow subsystem should oscillate to promote the switching between silence and fast oscillations in the fast subsystem. This, though, does not necessarily require a bidimensional slow subsystem. The slow oscillation, in fact, can occur through two mechanisms [17]:

- *Slow-wave burster*—The slow subsystem is a self-sustained oscillator, thus feedback from the fast to the slow subsystem is not required. In this case, the slow subsystem must be at least two-dimensional, $m\geqslant2$.
- *Hysteresis-loop burster*—The slow subsystem oscillates due to feedback from the fast subsystem. This can occur if the fast subsystem shows hysteresis between the silent and active states, which can be used to inform the slow subsystem about the state of the fast subsystem (e.g., by baseline). In this case one slow variable is enough, $m\geqslant1$.

Bursters come in different flavors. They can differ, among other factors, in the amplitude–frequency pattern of the active phase and in the behavior of the baseline. In the first formal classification of bursters, proposed by Rinzel [1], the author used these features to determine the bifurcations responsible for oscillations onset and offset in the fast subsystem of bursters found in biological systems. This type of classification based on the onset/offset bifurcations pair has been later systematically extended by Izhikevich in his seminal paper [17], with the goal of including not only the known bursters but also all the possible fast–slow ones. His classification includes 120 different pairs of onset/offset bifurcations, of which 16 pertain to a planar fast subsystem with a fixed point like silent state (resting-state could also be modeled with a small amplitude limit cycle). Izhikevich proposed to label each burster by stating the dimensionality of the fast and slow subsystem ($n+m$), the onset/offset bifurcations pair and whether the burster is of slow-wave or hysteresis-loop type.

In this work, we focus on bursters with the smallest dimensionality, namely $2+1$ for hysteresis loop and $2+2$ for slow wave. In both cases we have a planar ($n=2$) fast subsystem. In general, planar systems can exhibit only four codim-1 bifurcations (i.e. obtained by changing a single parameter) that allow the transition from a stable fixed point to a limit cycle, thus from the silent to the active phase. They are: saddle-node (SN), saddle-node-on-invariant-circle (SNIC), supercritical Hopf (supH) and subcritical Hopf (subH) bifurcations. Four bifurcations can be responsible for stopping the stable oscillation: SNIC, saddle-homoclinic (SH), supercritical Hopf and fold limit cycle (FLC). Considering all the pairs, we have sixteen different classes of planar bursters for slow-wave and sixteen for hysteresis loop [17].

**Fig. 2:**
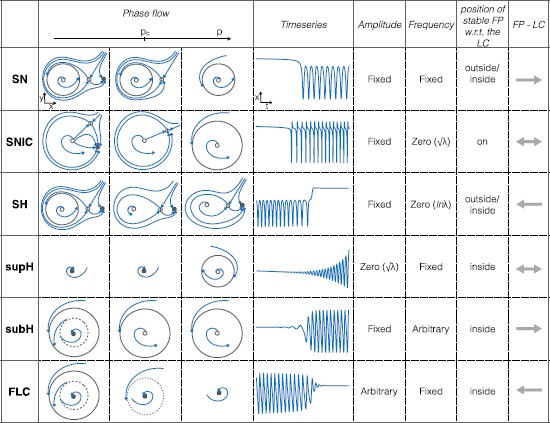
Six planar bifurcations are responsible for oscillation onset and offset. In a dynamical system, a bifurcation occurs when a smooth change of the values of some of the parameters of the system causes a sudden qualitative change of its behavior. The parameters that need to be varied to have this change in behavior are called bifurcation parameters. The number of bifurcation parameters necessary gives the *codimension* of the bifurcation. In planar systems, only six types of codim-1 bifurcations can cause the transition from silent (stable fixed point, FP) to oscillatory (limit cycle, LC) behavior and/or vice versa. Their characteristics are illustrated in this figure. For each bifurcation we report an example of how the state space changes when varying the bifurcation parameter *p*. Bifurcations occur at the critical value $p_{c}$. Stable, saddle, unstable fixed points are represented by *full*, *empty with a line inside*, *empty gray dots*, respectively. Stable, half-stable, unstable limit cycles are shown with *solid*, *dotted*, *dashed lines*, respectively. Orbits appear in *blue*. We also show an example of timeseries and report the amplitude–frequency behavior, where $\lambda=p-p_{c}$. In *the second last column* we state whether the stable fixed point is inside or outside the stable limit cycle. This can affect the behavior of the baseline in the timeseries. *The last column* indicates the reversibility of the bifurcation, in the direction of the FP to the LC

The six planar bifurcations are described in Fig. 2, with a sketch of the changes occurring in the state space when the bifurcation parameter varies, the corresponding timeseries, and the characteristic frequency-amplitude profiles [17]. Figure 2 also indicates whether the fixed point is encircled by the limit cycle (this can affect the behavior of the baseline), and whether a given bifurcation can be used at onset (→), offset (←) or both (↔) [17]. Tables 1 and 2 contain the abbreviations used in this paper. For brevity, we labeled the sixteen bursting classes by ‘c’ followed by a number from 1 to 16, in the order of appearance in Table 2. In the table we also report, when available, existing names in the literature for the classes.

**Table 1:** **Abbreviations**

Name	Alternative names	Abbreviation
*Codim-1 Bifurcations*		
Saddle-Node	Fold, tangential, limit point	SN
Saddle-Node on Invariant Circle	Saddle-node-loop of codim-1	SNIC
Hopf	Poincaré–Andronov–Hopf	H
Saddle Homoclinic	Saddle loop, homoclinic connection	SH
Fold Limit Cycle	Double cycle, fold of cycles, saddle-node of limit cycles, saddle node of periodic orbits	FLC
subcritical/supercritical		sub/sup
*Codim-2 Bifurcations*		
Cusp		C
Bautin	Degenerate Hopf–Takens, generalized Hopf	B
Takens–Bogdanov		TB
Degenerate Loop	Neutral-saddle-homoclinic loop	DL
Saddle-Node-Loop		SNL
*Codim-3 Bifurcations*		
degenerate Takens–Bogdanov		deg. TB
*Regions in the bifurcation diagram*		
Limit Cycle small—silent state outside the stable limit cycle		LCs
Limit Cycle big—stable limit cycle surrounds the silent state		LCb
*Other relevant points in the bifurcation diagram (not bifurcation points)*		
H and SN occur on two different fixed points		P_1,2_
FLC and SN occur for the same parameters’ values		P_3_

We report the abbreviations used in this manuscript for bifurcations and regions in the bifurcation diagram. For the bifurcations we report alternative names available in the literature.

**Table 2:** **Planar bursters**

Onset	Offset			
	SNIC	SH	supH	FLC
SN	c1	c2	c3	c4
	*Triangular*	*Square-wave*	*Tapered*	
		*Type I a, b*	*Type V*	*Type IV*
SNIC	c5	c6	c7	c8
	*Parabolic*			
	*Type II*			
supH	c9	c10	c11	c12
subH	c13	c14	c15	c16
				*Elliptic*
				*Type III*

The table shows the sixteen classes for planar bursters identified by Izhikevich. Rows give the onset bifurcations, columns the offset bifurcations. We labeled each class with a ‘c’ followed by a number from 1 to 16 as shown in the table. For each class we also report, when available, alternative names from the literature.

### The Unfolding Theory Approach

One of the goals of the present work is to find a minimal descriptive model for bursters with a planar fast subsystem, for simplicity called planar bursters. We adopt a strategy developed by Golubitsky et al. [19], based on earlier work by Bertram et al. [3] (see also de Vries [25]).

Bertram et al. used as fast subsystem a model with a two-parameter bifurcation diagram, the Chay–Cook model for pancreatic *β* cells bursting [28]. They located, in this two-parameter bifurcation diagram, horizontal cuts crossing the codim-1 bifurcation curves required for some of the bursting classes known at that time. Horizontal cuts are straight paths in the parameter plane along which only one parameter is changing. This parameter is then used as slow variable. Using the same model, they could produce different classes by changing the location of the cut in the two-parameter bifurcation diagram.

This strategy has been later formalized by Golubitsky et al. [19]. They realized that the codim-1 bifurcations of the fast subsystem which are necessary for bursting can be collapsed to a single local singularity of higher codimension, that is, a singularity in a high-dimensional parameter space, where the codim-1 bifurcation curves coincide. A path for bursting activity can then be found in the so-called unfolding of the singularity.

The unfolding of a singularity of a dynamical system is a system that exhibits all possible bifurcations of that singularity [29]. This unfolding can be described by adding some terms containing extra parameters to the normal form of the singularity. The number of extra parameters necessary, called *unfolding parameters*, is the codimension of the singularity. In the unfolding parameter space there are manifolds (e.g. curves, surfaces) of lower codimension bifurcation points. These manifolds intersect at the origin, that is, where all the extra parameters are zero and the system is equal to the normal form of the singularity. In the unfolding, we can search for paths that cross the right sequence of codim-1 bifurcations required by the burster, as done by Bertram et al. in the two-parameter bifurcation diagram of the Chay–Cook model.

Let us consider the subH/FLC burster, for instance. To have hysteresis for this class, no additional bifurcations, apart for those at onset and offset, are required. We can thus take the unfolding of the codim-2 Bautin (also known as degenerate Hopf) singularity at which fold limit cycle and Hopf bifurcations occur together. In the unfolding, a curve of fold limit cycle bifurcations and a curve of Hopf (divided in a supercritical and a subcritical branch) stem from the Bautin point. We can thus locate a path for subH/FLC bursting. The path does not need to be horizontal, as long as it can be parametrized in terms of the slow variables. In this case, having hysteresis, one slow variable is enough.

The advantage of this approach is that we can use normal forms for the unfolding, if available, providing a minimal description for the fast subsystem.

Golubitsky and coworkers systematically investigated the unfoldings of codim-1 and codim-2 bifurcations, with respect to bursting paths. They also extended the work to some regions close to a codim-3 singularity, but in a non-complete fashion. With regard to bursters with a planar fast subsystem, they identified nine slow-wave and three hysteresis-loop bursters. The hysteresis loop can be harder to locate because, to exhibit hysteresis, they may require more bifurcations than their slow-wave counterpart. For example, consider the supH/supH burster, two supercritical Hopf bifurcations alone are not enough to create hysteresis, but the slow-wave burster can be built by going back and forth through a single supercritical Hopf point. On the other hand, hysteresis-loop bursters have a simpler mechanism than slow-wave bursters, with regard to the slow dynamics. In slow-wave bursting the slow subsystem must be at least two-dimensional and the path to follow in the unfolding must be completely specified. Hysteresis-loop bursting can be obtained with just one slowly changing variable and it is enough to specify the curve on which the path has to lie and the orientation, while the points at which *z* inverts its direction over the course of time are determined by the crossing of the onset and offset bifurcation manifolds.

### Codim-3 Degenerate Takens–Bogdanov Singularity

The codim-3 singularity used by Golubitsky et al. is called degenerate Takens–Bogdanov (deg. TB). Four topologically different unfoldings of this singularity have been identified by Dumortier et al. [22, 23]. These unfoldings are very rich, containing saddle-node, SNIC, saddle-homoclinic, supercritical Hopf, subcritical Hopf and fold limit cycle bifurcations [23]. It has been proposed that the unfolding of this singularity could provide a minimal model to understand neuronal excitability and its modulation [30, 31], or a qualitative model for a cortical mass [32]. In addition, the deg. TB singularity has appeared when investigating some of the models for neural bursting [3, 25] and its biological importance for bursting has been further underlined by Osinga et al. [20]. In one of the unfoldings of the deg. TB, the authors identified paths for many known bursters related to cell activity. They also implemented a slow-wave bursting model.

In the present work we systematically extend the work by Golubitsky et al. to the deg. TB singularity and investigate its four unfoldings. We aim at uncovering the presence or absence not only of paths for bursters known from cell activity, but of all planar bursters present in Izhikevich’s classification. This would provide a general model ready to be applied in cell bursting and in any other field for which bursting classification is in progress, such as epileptic seizure modeling [15]. We give indications on how to build slow-wave bursters, which is in line with the work of [20]. Furthermore we make use of hysteresis, when present, to build hysteresis-loop bursters. This allows less constraints on the required path and make it simpler to implement transitions between different bursting classes (see also Franci et al. [21]).

A description of the planar codim-3 deg. TB singularity’s equations and unfoldings has been provided by Dumortier et al. [22, 23]. They identified four topologically different possibilities for this singularity and referred to them as codim-3 deg. TB cases: cusp, saddle, focus and elliptic. We investigated the three-parameter unfoldings of all these cases looking for possible paths for bursting activity, considering both the time forward ($t\rightarrow\infty$) and the time reversed conditions ($t\rightarrow-\infty$).

We found that the deg. TB singularity for the focus case in time reversed condition gives the largest amount of bursting paths. Exploring the other cases did not result in a description of new classes. For this reason, Sect. 2.5 is devoted to a detailed description of the focus case unfolding. In Sect. 3 we recall the presentation of the bursting classes identified in this unfolding by Bertram et al. [3] (see also the appendix in [20]) and we identify new classes. We then use this description to build a single model, which is able to display a vast repertoire of bursting activities.

Results for the focus case in time forward condition and for the cusp, saddle, and elliptic cases are briefly summarized in Sect. 3.6. More details are provided in Sects. 5.7 and 5.8.

### Unfolding the Deg. TB Singularity of Focus Case

The unfolding of the focus case in the time reversed condition is described by the following system of two coupled state variables $( x,y )$ [23]:

2 $$ \textstyle\begin{cases} \dot{x}=-{y,}\\ \dot{y}=x^{3}-\mu_{2} x-\mu_{1}-y(\nu+b x+x^{2}), \end{cases} $$

where the dot above a variable describes the time derivative $d/dt$ and $( \mu_{1}, \mu_{2},\nu )$ are the three unfolding parameters. The unfoldings obtained for any value of *b* within the interval $0< b<2\sqrt{2}$ are topologically equivalent and correspond to the focus case [23]. In the present work, we can thus set $b=1$ without any loss of generality. When $b>2\sqrt{2}$, instead, Eq. (2) describes the elliptic case. Topological equivalence between the focus and elliptic cases has been shown by Baer et al. [24], which we will address in more detail in Sect. 3.6.

In the following three subsections we will discuss how changes in the unfolding parameters $( \mu_{1},\mu_{2},\nu )$ affect the state space spanned by the variables $( x,y )$. These results, including a complete analysis of the unfolding and its bifurcations, have been previously reported by Dumortier et al. [23] and we include them here for completeness.

#### Representation on a Sphere

The codim-3 bifurcation occurs when the three unfolding parameters are equal to zero. Here saddle-node, Hopf, SNIC, saddle-homoclinic and fold limit cycle bifurcations coincide. From the origin of the parameter space, surfaces for codim-1 bifurcations arise. At the intersection between surfaces of codim-1 bifurcations we have curves of codim-2 bifurcations.

**Fig. 3:**
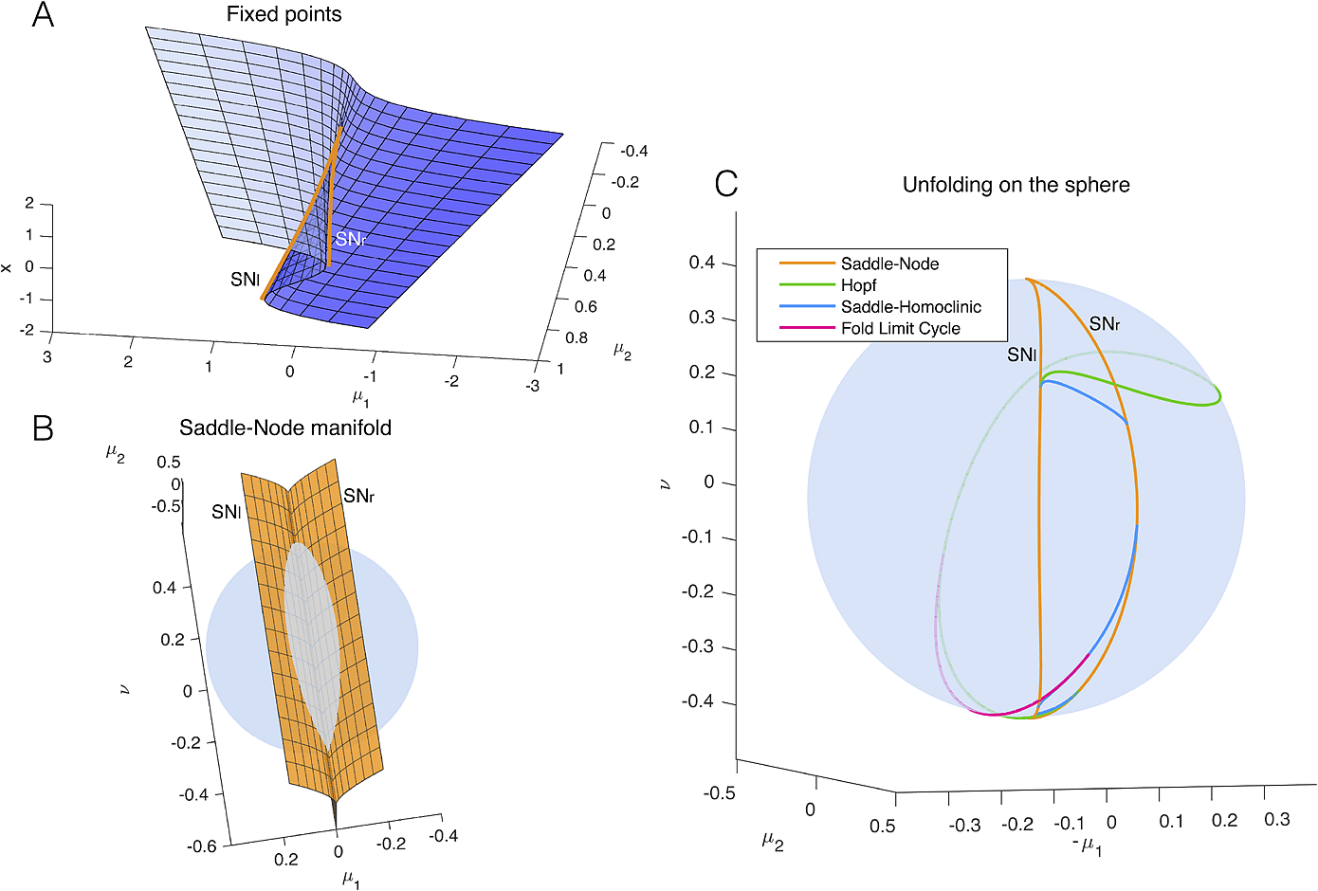
Unfolding of the deg. TB singularity, focus case. **A** The fixed points of the system are found for $y_{0}=0$, $x_{0}^{3}+\mu_{2} x_{0}-\mu _{1}=0$. *The blue surface* represents $x_{0}$ plotted against the two parameters $\mu_{1}$, $\mu_{2}$. In *orange* are marked the curves of saddle-node bifurcations at which the saddle solution (i.e. the middle branch) collides with the focus in the upper or lower branch and annihilates. **B** The manifold of the saddle-node bifurcation (in *orange*) is plotted in the three dimensional unfolding parameters space together with a sphere of radius $R = 0.4$ centered at the origin of the parameter space $(\mu_{1},\mu_{2},\nu)=(0,0,0)$. The intersection between the surface of saddle-node bifurcations and the spherical surface gives a curve of saddle-node bifurcation. **C** The bifurcation curves obtained at the intersection between the sphere and all the bifurcation surfaces of the unfolding. In this figure we do not separate between supercritical and subcritical Hopf (both in *green*) and between saddle node and SNIC (both in *orange*)

**Fig. 4:**
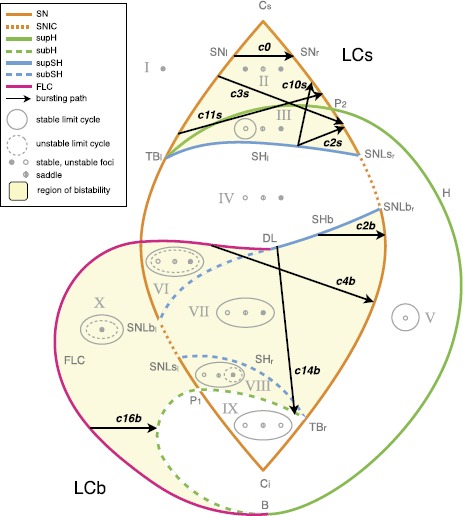
Paths for hysteresis-loop bursting activity in the unfolding of the deg. TB singularity of focus type. This is a flat representation topologically equivalent to the bifurcation diagram shown in Fig. 3C. Different bifurcation curves are indicated with different *colors*, as indicated in *the legend*. *The subscripts l, r, s, i* (left, right, superior, inferior) refer to where, in the state space, the bifurcation occurs. For each region of the diagram, labeled with *a Roman numeral from I to X*, a schematic description of the phase portrait is proposed in *gray*, using *solid/dashed circles* to represent stable/unstable limit cycle, *full/empty dots* for stable/unstable foci and *an empty dot with a line in the middle* to represent saddles. A more detailed description of these ten regions is given in Fig. 5. There are two separate regions of bistability (in *yellow*): one, labeled LCb (limit cycle big), in the lower part of the unfolding, where the stable limit cycle is big enough to surround all the fixed points existing; the other, labeled LCs region, in the upper part, where the limit cycle does not surround all the fixed points. Paths for bursting activity are drawn as *black arrows*. The direction is chosen so that the path encounters first the offset and then the onset bifurcations. When building the model, this will be the direction along which the slow variable increases

**Fig. 5:**
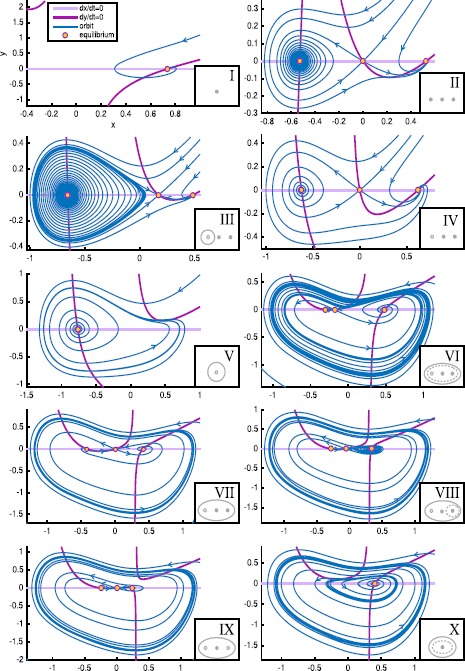
Phase flows. For each region of the unfolding, labeled with a Roman numeral in Fig. 4, we show the nullclines of Eq. (2) in *light and dark purple*. Fixed points at the intersection of the nullclines are marked with *yellow dots*. Flows are shown in *blue*. Nullclines and fixed points are computed analytically, flows are obtained through numerical simulations in Matcont

To describe what happens in the neighbourhood of the codim-3 singularity we can consider the intersection of the bifurcation curves and surfaces with a sphere centered around the singularity at the origin. On the sphere we have curves of codim-1, points of codim-2 and no codim-3 bifurcations. The bifurcation portrait on the sphere is topologically equivalent for any sufficiently small value of the radius *R* [23]. For larger radii the topological equivalence is not guaranteed and Eq. (2) does not describe the unfolding of the deg. TB singularity anymore. Using a fixed radius allows for a description of the bifurcations with two parameters, that is, the spherical coordinates $( \theta,\phi )$, instead of three parameters $(\mu_{1},\mu_{2},\nu)$ in Cartesian coordinates. The result of the numerical evaluation of the unfolding on the sphere that we reproduced from the literature [20, 23] is shown in Fig. 3C. The details, including all labeled bifurcations, are presented in the topologically equivalent flat sketch in Fig. 4, for easier reading (see also [23]). Bifurcation curves divide the space in different regions, labeled with Roman numerals. The state space for each region is qualitatively sketched in gray. In Fig. 4 are also present black arrows, which represent possible bursting paths as will be explained in Sect. 3.1. State spaces for the different regions labeled with Roman numerals are fully described in Fig. 5. Nullclines for *x* and *y* are shown in light and dark purple respectively and fixed points with yellow circles. In blue there are orbits indicating the flow. More details can be found in Sect. 5.2.

#### Fixed Points and Local Bifurcations

The system is in a fixed point, or equilibrium, $(x_{0}, y_{0})$ when invariant with respect to time t, that is, $\dot{x}=0$, $\dot{y}=0$. The corresponding solution for Eq. (2) is $y_{0}=0$, $x_{0}^{3}+\mu_{2} x_{0}-\mu_{1}=0$. Hence, the fixed points do not depend on *ν*. $x_{0}$ is displayed in Fig. 3A as a function of $(\mu_{1}, \mu_{2})$. We can distinguish two regions in the space $(\mu_{1}\ \mu_{2})$: one in which a single fixed point, a focus, exists; the other in which we have a focus on an upper branch, a focus on a lower branch and a saddle on a middle branch. The saddle coalesces with the focus of the upper branch along the saddle-node bifurcation curve SN_*r*_ and with the focus of the lower branch along SN_*l*_. Right and left (and later inferior, superior) refer to where the bifurcation occurs in the state space, as proposed in [23]. Figure 3B shows the saddle-node bifurcation in the complete parameter space of the unfolding and its intersection with the sphere: a closed curve which delimitates the region with three fixed points from the region with a single fixed point. The two saddle-node bifurcation surfaces, SN_*l*_ and SN_*r*_, meet along a line of codimen-2 cusp points. This line intersects with the sphere in two points, labeled in Fig. 4 as C_*s*_ and C_*i*_.

The condition for the Hopf bifurcation can be found by equating the trace of the Jacobian (at the fixed point) to zero. The Hopf bifurcation takes place if $x^{2}+x+\nu=0$ and $x^{3}-\mu_{2} x-\mu_{1}=0$. The Hopf bifurcation in the unfolding is represented by green lines in Fig. 3C and Fig. 4, where a solid/dashed line is used for the supercritical/subcritical cases. On the sphere, we have two codim-2 Takens–Bogdanov bifurcation points where the Hopf and saddle-node bifurcations, TB_*l*_ on SN_*l*_ and TB_*r*_ on SN_*r*_, meet. Note that the other two intersections between the Hopf and saddle-node bifurcation curves in Fig. 4, P_1_ and P_2_, are not Takens–Bogdanov points as the two bifurcations act on two different foci.

The saddle-node and the Hopf bifurcations just described are the local bifurcations in this unfolding, that is, the bifurcations changing the stability of fixed points.

#### Global Bifurcations

Results for the global bifurcations, affecting broader regions of the state space, are here obtained numerically and discussed analytically in [23]. At most one stable limit cycle exists in the system given by Eq. (2). To describe the unfolding, we can consider the stable limit cycle originating at the supH curve, between TB_*l*_ and the Bautin point B, and we can follow its evolution and annihilation. Starting at the TB_*l*_, the limit cycle arises from the destabilization of the stable focus on the lower branch and grows until it meets the saddle in the middle branch. Here the limit cycle vanishes and we have a curve of saddle-homoclinic bifurcations SH_*l*_, which starts at TB_*l*_ and terminates on SN_*r*_ giving rise to the codim-2 saddle-node-loop bifurcation SNLs_*r*_. ‘s’ denotes a small limit cycle, in the sense that it does not surround all the fixed points. From SNLs_*r*_ to SNLb_*r*_, the limit cycle disappears through a SNIC bifurcation giving rise to a heteroclinic trajectory between the saddle and the stable focus appeared through SN_*r*_. SNLb_*r*_ marks the point where the limit cycle has grown big enough to encircle all the fixed points. From here to the DL (degenerate loop) point, in fact, the limit cycle disappears through a ‘big saddle-homoclinic’ bifurcation SHb (a saddle-homoclinic bifurcation is said ‘big’ if the saddle’s unstable manifold returns to the saddle along the other direction of the saddle’s stable manifold [17, 33], this implies here that the limit cycle encompasses the stable fixed point). After DL, the limit cycle is not able to reach the saddle anymore and coalesces with an unstable limit cycle on the fold limit cycles curve FLC. This unstable limit cycle, which is always enclosed by the stable one, can originate in two ways: from the subcritical branch of the Hopf curve or from the subcritical branch of SHb. The unstable limit cycle can also disappear before reaching the FLC curve, via a SNIC bifurcation, from SNLs_*l*_ to SNLb_*l*_.

## Results

### Hysteresis-Loop Bursting Classes

We investigated the two-parameter bifurcation topology (i.e. the unfolding on the sphere) to identify paths for bursting activity. Following [19, 20], the system given in Eq. (2) can be considered as the fast subsystem. We then used a one-dimensional slow subsystem to slowly steer the fast subsystem in the parameter space so that bursting behavior can occur.

In the present work we are particularly interested in bursters driven by a single slow variable, which oscillates due to feedback from the fast subsystem. For this purpose, the state space of the fast subsystem must display hysteresis between the silent and the active states. The slow variable can be instructed, in the simplest form by linear feedback, to steer the path in a given direction when the system is close to a stable fixed point representing the silent phase, and in the opposite direction when the system has moved to another attractor and is thus far from the silent phase. If this second attractor is a limit cycle, the system is in the active phase.

A prerequisite of hysteresis is the existence of a regime in which at least two stable states coexist, that is, bistability. We find two regions on the sphere where bistability occurs (in yellow in Fig. 4). One region is in the lower portion of the bifurcation diagram, where the limit cycle surrounds the fixed point that acts as silent state, which we named LCb (Limit Cycle big) region. The other region is in the upper part of Fig. 4, here the silent state is outside the limit cycle. We named it LCs (Limit Cycle small) region. We added ‘b’ or ‘s’ to the labels of bursting classes to identify the region where they occur.

#### LCs Bursters

In the region LCs, oscillations can start through the SN bifurcation (SN_*r*_ between SNLs_*r*_ and P_2_) or the supH. The limit cycle can vanish through the supHopf or the SH bifurcations. Consequently, we considered and verified the existence of four pairs of onset/offset bifurcations: c2s (SN/SH), c3s (SN/supH), c10s (supH/SH) and c11s (supH/supH). Among these classes, to the best of our knowledge, c11s was not previously identified in this unfolding (see references [3, 20, 25] and Sect. 4 for the other classes). This region contains in addition to these four cases a special case of burster in which no limit cycle exists and both active and silent phases are given by fixed points (point-point burster [17]). In this case both onset and offset are given by the SN bifurcation. When the stable focus, which represents the silent phase, destabilizes, the system spirals towards the other stable focus. This spiraling is the active phase. We attributed the number 0 to the SN/SN bursting class, which is not among the sixteen point-cycle classes.

**Fig. 6:**
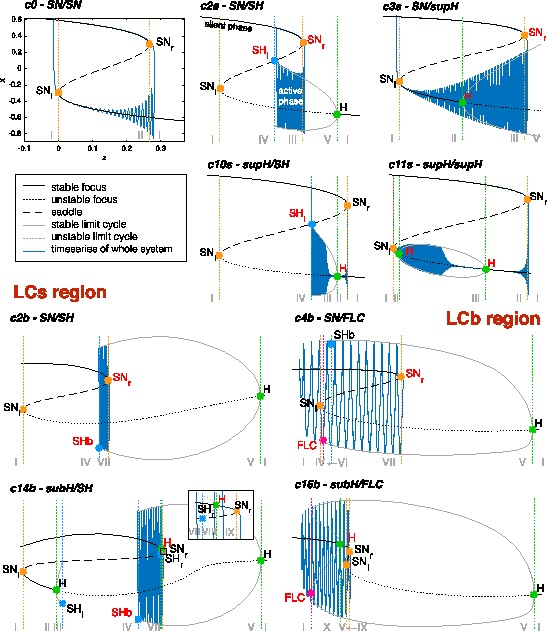
Bifurcation diagrams along bursting paths. For each bursting class found in the unfolding we produced the bifurcation diagram of the fast subsystem using *z* as bifurcation parameter and plotting the fast variable *x* as ordinate. The simulated bursting trajectory, in *blue*, is superimposed to the bifurcation diagram. Bifurcations are marked with *a colored star* (same color code as in Fig. 4) and *vertical dotted line of the same color* separate regions of the unfolding. *The Roman numeral* of the relative region is written in *gray in the lower part of the plots*. Onset and offset bifurcations are written in *red*. In all the nine classes (eight point-cycle and one point-point bursters) the upper branch of the z-shaped curve of fixed points acts as silent state. When the fast subsystem is in the silent state, the slow variable *z* is instructed to increase. At SN_*r*_ (or at the subcritical Hopf point in classes c14b and c16b) the resting-state destabilizes, the system moves towards another attractor, and *z* start decreasing until the system goes back to the silent state and a new bursting cycle is started. The active phase takes place on the limit cycle surrounding the lower branch of the equilibrium manifold. *The top panel* shows bifurcation diagrams for bursters in the LCs region, *the bottom panel* for those in the LCb region. In the LCs region we can have a saddle-node onset if the limit cycle exists already when the silent state destabilizes at SN_*r*_ (*first row*) or supercritical Hopf onset when the limit cycle is created for smaller values of *z* (*second row*). Oscillations can end because the limit cycle coalesces with the saddle (*middle column*) or because of another supercritical Hopf bifurcation (*right column*). In *the LCb region panel*, in *the first row* oscillations are started through saddle-node bifurcation, while the resting-state destabilizes earlier in *the second row* via subcritical Hopf bifurcation. The limit cycle coalesces with the saddle in *the left column* and with an unstable limit cycle in *the right column*. Bursting paths and entire arcs of great circles used to produce these bifurcation diagrams are shown in Sect. 5.3

An example of a path for each class is indicated by a black arrow in Fig. 4. Unless otherwise specified, we verified that each bursting path could be obtained with an arc of great circle on the sphere crossing the offset and onset bifurcations, together with other bifurcations that may be needed to close the hysteresis loop. In the cartoon unfolding in Fig. 4 these paths are simply represented by straight arrows. In Sect. 5.3 we provide more details of the requisites that each path has to satisfy, and show a projection of the real paths used in Sect. 3.2 for the simulations of the model. The top panel of Fig. 6 shows bifurcation diagrams relative to these paths. They are obtained by parametrizing each path in terms of a parameter *z* (which will later be used as slow variable) and considering the latter as the bifurcation parameter. We superimposed the simulated bursting trajectories (obtained through the model that will be described in the next section) in blue. Stars mark the occurrence of bifurcations, with the same color code as in Fig. 4. Bifurcations separate different regions of the unfolding traversed by the path, Roman numerals relative to each region are reported in the lower part of the plot.

#### LCb Bursters

In the LCb region, oscillations can be generated by the SN bifurcation (SN_*r*_) or the subHopf. Oscillations can be stopped through the SH or the FLC bifurcations. Consequently, we considered and verified the existence of classes given by four pairs of onset/offset bifurcations: c2b (SN/SH), c4b (SN/FLC), c14b (subH/SH) and c16b (subH/FLC). Class c14b was not identified before in this unfolding, while the others have been reported in [3, 20, 25].

Examples of paths are shown in Fig. 4, and bifurcation diagrams are shown in the bottom panel of Fig. 6, more details are in Sect. 5.3.

### Hysteresis-Loop Bursters: A Unique Model

Equation (2) is the canonical form of the unfolding of the codim-3 deg. TB singularity, focus case, with time reversed [23] and is reproduced here with $b=1$ for convenience:

3 $$ \textstyle\begin{cases} \dot{x}=-{y,}\\ \dot{y}=x^{3}-\mu_{2} x-\mu_{1}-y(\nu+x+x^{2}). \end{cases} $$

We use these equations to describe the fast subsystem. For bursting activity we need the fast subsystem to slowly move in the unfolding parameter space following a path to undergo the required bifurcations. We can parametrize this path in terms of a third variable *z*, which slowly changes in time. This variable steers the system through the parameter space and drives it into and out of oscillatory behavior. With reference to Eq. (1), this implies $\mathbf {x}=(x,y)$ and $\mathbf{z}=z$.

In particular, when the distance in phase space between the state of the fast subsystem $( x,y )$ and the silent state $(x_{s}(z),0)$ is smaller than a certain threshold $d^{*}$ the system should move to the point in parameter space where the silent state loses stability. On the other hand, when the distance between the state of the system and the silent state is bigger than $d^{*}$ the system should move to the point in the parameter space where the limit cycle destabilizes. In other words, when the system is silent, it has to move in the direction of the bursting onset bifurcation, when it is active, it has to move towards the bursting offset bifurcation. If we consider a curve in the parameter space, which starts at the offset bifurcation and extends towards the onset bifurcation (as indicated by the black arrows in the bifurcation diagrams in Fig. 4), *ż* should be positive when the system is in the silent state ($\sqrt {(x-x_{s}(z))^{2}+y^{2}}< d^{*}$) and negative otherwise. This choice is arbitrary. One could also consider a path going from the onset point to the offset one and invert the behavior of *z* (decreasing/increasing when the fast system is in the silent/active state respectively). With our choice, the temporal dynamics of *z* can thus be described by

4 $$ \textstyle\begin{cases} \dot{x}=-{y,}\\ \dot{y}=x^{3}-\mu_{2}(z) x-\mu_{1}(z)-y(\nu(z)+x+x^{2}),\\ \dot{z}=-c(\sqrt{(x-x_{s}(z))^{2}+y^{2}}-d^{*}), \end{cases} $$

where *c* is the velocity at which *z* changes along the path.

As described in [23] and summarized in Sect. 2.5, the unfolding parameters can be reduced to two if we restrict the movements to a spherical surface centered at the codim-3 singularity. We can perform this reduction without loss of generality because the bifurcation curves on the sphere will be topologically equivalent to those on any other sphere, providing a small enough radius.

With the coordinate transformation for the unfolding parameters used in [23] and described by

5 $$ \textstyle\begin{cases} \mu_{2} = R\sin{\theta}\cos{\phi},\\ \mu_{1} = -R\sin{\theta}\sin{\phi},\\ \nu=R \cos{\theta}, \end{cases} $$

the model in Eq. (4) reads

6 $$ \textstyle\begin{cases} \dot{x}=-{y},\\ \dot{y}=x^{3}-R \sin{\theta(z)}\cos{\phi(z)} x+R\sin{\theta(z)}\sin{\phi (z)}\\ \hphantom{\dot{y}=}-y(R\cos{\theta(z)}+x+x^{2}),\\ \dot{z}=-c(\sqrt{(x-x_{s}(z))^{2}+y^{2}}-d^{*}). \end{cases} $$

The simplest curve satisfying the requirements, considering that our two-dimensional parameter space lies on a spherical surface, is the shortest arc on this surface between the initial and final point, known as great circle.

To provide a parametrization of the great circle we consider a circumference of radius *R*, with center in the origin and passing through the points described by the Cartesian vectors **A** and **B**. The parametric equations of this circumference for the vector of Cartesian coordinates

$$\boldsymbol{\mu}= \begin{pmatrix}\mu_{2}\\-\mu_{1}\\\nu \end{pmatrix} $$

are

7 $$ \boldsymbol{\mu}(z)=R (\mathbf{e}\sin{z}+\mathbf{f}\cos{z}), $$

where $\mathbf{e}=\mathbf{A}/R$ and $\mathbf{f}=((\mathbf{A}\times \mathbf{B})\times\mathbf{A})/\Vert(\mathbf{A}\times\mathbf{B})\times \mathbf{A}\Vert$ (× is the cross product and $\Vert\text{ }\Vert$ the L2-norm) are chosen so that the path starts at **A** and moves towards **B**. The parametric equations of the corresponding great circle in spherical coordinates are thus

8 $$ \textstyle\begin{cases} r(z)=R,\\ \theta(z)=\arccos (\frac{\nu(z)}{R} ),\\ \phi(z)=\arctan (\frac{-\mu_{1}(z)}{\mu_{2}(z)} ). \end{cases} $$

This parametrization (Eqs. (7)–(8)) and Eq. (6) provide a model able to reproduce all hysteresis-loop bursting classes found in the unfolding of the deg. TB singularity, focus case.

To summarize the elements appearing in the model, we have a fast subsystem $( x,y)$, which in some regions of the unfolding space presents bistability and hysteresis between a stable fixed point and a stable limit cycle; and a slow subsystem *z*, which depends on the feedback from the fast subsystem and moves the latter through the parameter space. When the fast subsystem shows hysteresis, the slow one can drive it in and out from the oscillatory behavior giving rise to bursting activity. The constant *c* determines the speed of the movement of the subsystem in the parameter space, as promoted by *z*. It should be small enough to guarantee time-scale separation between the fast and slow subsystems. This constant affects the length of both silent and active phases: the slower the movement the bigger the number of oscillations in the active phase (under the condition that the other parameters are fixed). The role of the silent state is played by the upper branch of the equilibrium manifold, of state coordinates $(x,y)=(x_{s}(\theta(z),\phi(z)),0)$.

**Fig. 7:**
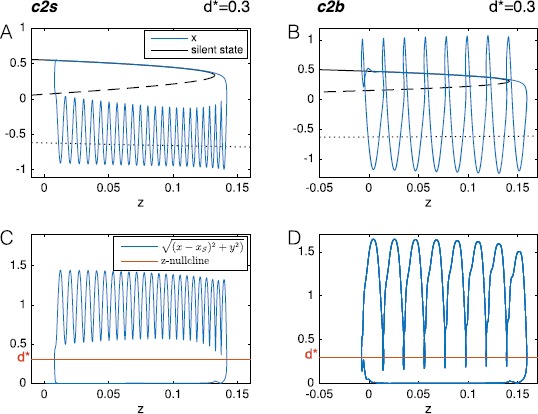
Slow dynamics. We choose class SN/SH c2s (*left panel*) as an example for classes in which the silent state is outside the limit cycle. In **A** it can be observed that the projections of the oscillations on *the* *x-axis* can be separated from that of the silent state. This is not true for classes in which the silent state is encircled by the limit cycle, as in class SN/SH c2b (*right panel*). For this class the projection of the oscillations on *the* *x-axis* cannot be separated by that of the silent state (**B**). This separation becomes possible if we use, instead of the *x*-variable of the bursting trajectory, the distance of the latter from the silent state ($\sqrt{(x-x_{s})^{2}+y^{2}}$) (**C**–**D**). In **C** and **D** we added in *red* the *z*-nullcline

For the dynamics of the slow variable, a common choice when designing bursters is to let *ż* depend linearly on one of the fast variables [34]. This approach would work for the classes in LCs, where the silent state is outside the stable limit cycle. In this case *z* can be instructed to increase/decrease depending on whether *x* is close/far from the silent state, as we can find an axis along which the projections of the fixed point and limit cycle are separated. An example would be the class SN/SH c2s, as shown in Fig. 7A, where silent state and limit cycle are separated when projected on the *x*-axis. On the other hand, if the silent state is inside the stable limit cycle, as for the classes in the LCb region, this mechanism does not hold anymore, because during the oscillatory phase *x* would periodically get close, or be equal, to $x_{s}$. See, for example, Fig. 7B for the class SN/SH c2b. A solution could be to introduce in the slow dynamics a function that computes the average of the past activity, for example the mean of the oscillations if this differs from the value of the silent state [34]. Another possibility, which avoids the integration over past states of the system, is to transform $(x,y)$ to complex [35] or polar coordinates. Values of the module or radial coordinate of the limit cycle are now separated from those of the silent state. We can thus build a linear dynamics depending on the module or radial coordinate. This is our choice, and given that the position of the silent state can vary depending on the position in the unfolding, we decided to have a linear dynamics depending on the distance to the silent state. This ensures that the same slow equation can produce bursting activity in different regions of the unfolding. While in the first row of Fig. 7 we plot the *x* coordinate of the bursting trajectory for the two classes, in the second row we show the distance between this trajectory and the silent state. Note that also for c2b it is now possible to separate the oscillatory state from the silent one. We added the *z*-nullcline in red ($d^{*}=\sqrt{(x-x_{S})^{2}+y^{2}}$). The parameter $d^{*}$ is the threshold that determines the distance of the fast subsystem from the silent state required to promote a change in the direction of *z*. It should not exceed the mean distance between the silent state and the limit cycle. The smaller it is, the bigger is the silent/active phase lengths ratio. Bursting does not occur for $d^{*}\leq0$. When $d^{*}<0$ there is no intersection between the *z*-nullcline and the branch of silent states (which in Fig. 7C, D corresponds to $\sqrt{(x-x_{S})^{2}+y^{2}}=0$). When $d^{*}=0$ the *z*-nullcline lies on the silent state and the whole system is in a stable fixed point.

Once the slow dynamics is defined, what differentiates among classes is the location of the points **A** and **B**. We confined the movement to a sphere of radius *R* in the parameter space $(\mu_{2}, -\mu_{1}, \nu)$ centered on the codim-3 singularity at the origin. We fixed $R=0.4$ in this work, unless otherwise specified. Points **A** and **B** determine the great circle on which the arc has to lie and the direction of movement (from **A** to **B**). The initial point of the path is **A**. The mechanism that forces *z* to change direction over the course of time will automatically set the final point of the path. In the following we will choose **B** so that it lies on the last bifurcation curve encountered by the system before *z* changes direction.

**Fig. 8:**
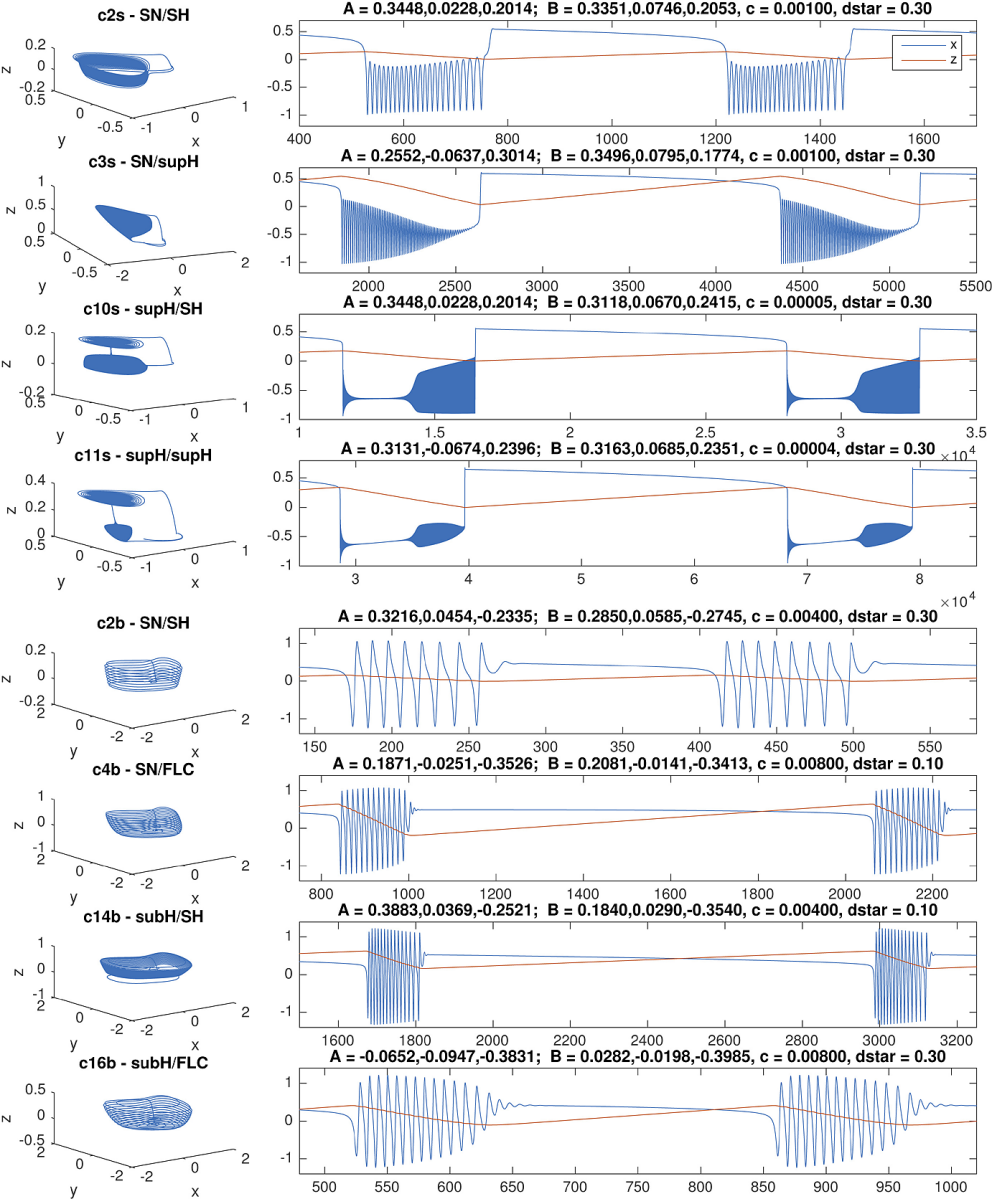
Timeseries of the hysteresis-loop bursting classes. For each of the classes the evolution is portrayed in the phase space (*left panel*) and the timeseries for *x* and *z* (*right panel*) are plotted. To obtain different classes we tuned the parameters **A** and **B** which affect the position of the path in the unfolding. The corresponding parameter values are reported *above each plot*

We performed simulations for each of the classes found in the focus case, as shown in Fig. 8. For each class, the evolution in the phase space is also shown.

**Fig. 9:**
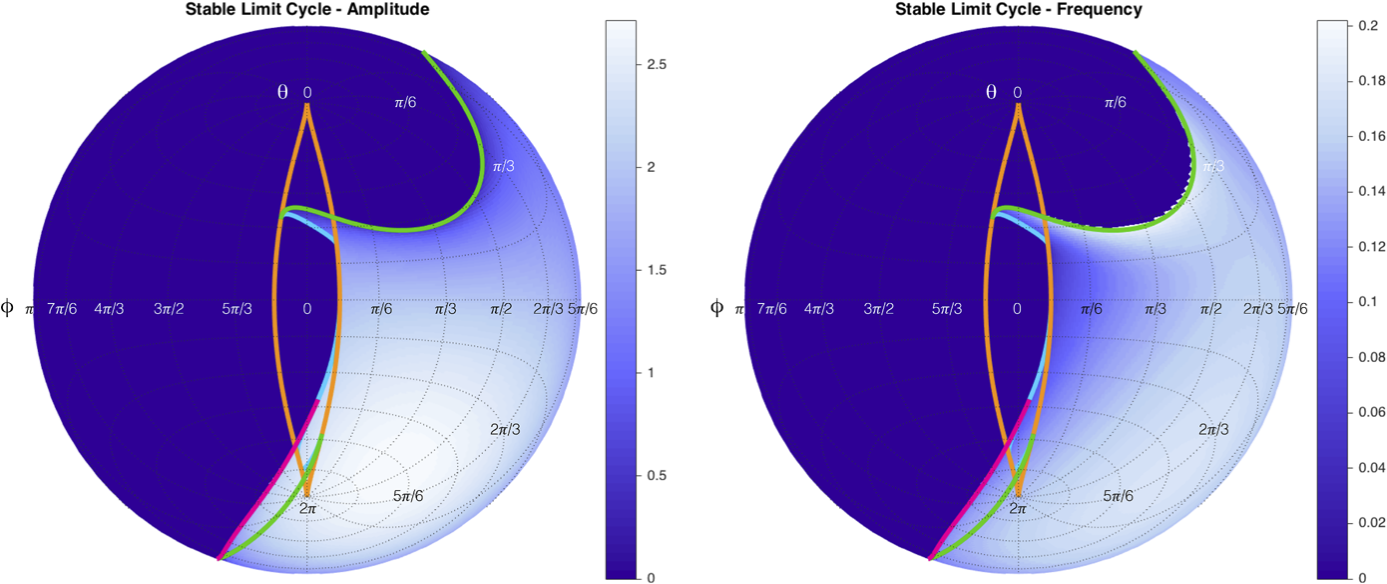
Stable limit cycle of the focus case. We investigated the amplitude (*left panel*) and frequency (*right panel*) of the single stable limit cycle present in the focus case. The frequency is measured in Hz. To obtain a flat representation of the spherical surface we make use of Lambert equal area azimuthal projection

The same class can be obtained in different ways, by changing the location of the points **A** and **B**. To explore the effects of the location of the path on the shape of the timeseries, we investigated how the amplitude and frequency of the stable limit cycle change across the unfolding. Given that both frequency and amplitude in this unfolding are non-local properties, influenced by both local and global bifurcations, we obtained these results numerically. We performed simulations at different points on the sphere of radius *R*, and computed amplitude and frequency from the timeseries (see Sect. 5.2 for details). Results are shown in Fig. 9. From the figure it is possible to appreciate the effect of the presence of different bifurcations. Infinite period bifurcations (such as SNIC or SH), for example, create gradients in the frequency plot while the supH bifurcation brings the amplitude to zero. By choosing different paths it is thus possible to alter the amplitude–frequency profile of timeseries, within the constraints imposed by a given class.

**Fig. 10:**
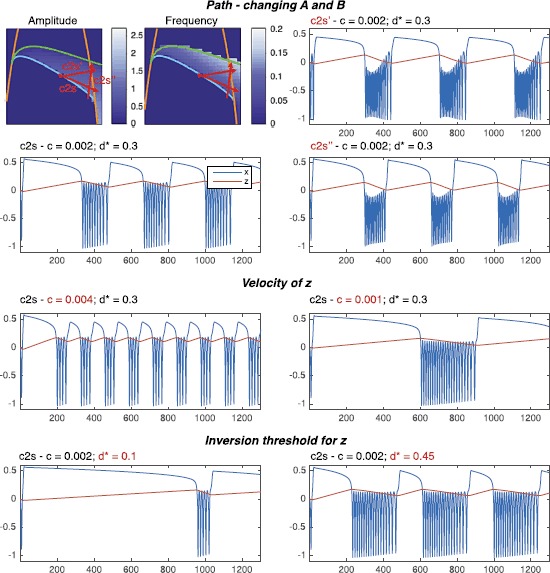
Effect of the parameters of the model on the timeseries of a class. We show how different realizations of the same class can be obtained by changing the parameters of the model. We use as example c2s (SN/SH). In *the top panel* we show how the amplitude–frequency profile of a given class can be changed by choosing different paths (c2s, c2s’, c2s”) in the unfolding. The only requirement is that the starting point **A** of the path has to lay on the SH_*l*_ curve and the ending point **B** on SN_*r*_ curve between SNLs_*r*_ and $p_{1}$. In *the middle panel* we show how the velocity of *z*, the parameter *c*, affects the length of both the active and the silent phases. In *the bottom panel* we can see how changing $d^{\star}$ affects the active/silent phases ratio. The values for **A** and **B** used to obtain the simulations are: for c2s $\mathbf {A}=[0.3448,0.02285,0.2014]$, $\mathbf{B}=[0.3496,0.07955,0.1774]$; for c2s’ $\mathbf{A}=[0.3448,0.02285,0.2014]$, $\mathbf {B}=[0.3331,0.074,0.2087]$; for c2s” $\mathbf {A}=[0.3551,0.07019,0.1703]$, $\mathbf{B}=[0.3331,0.074,0.2087]$

As an example, we show in Fig. 10 three different realizations for the class c2s. While all three realizations have a decreasing frequency towards bursting offset, as prescribed by the SH bifurcation, the amplitude behavior may vary (increasing, for example, in c2s’, more constant in c2s and c2s”). In the same figure we also show the impact of varying the velocity, *c*, at which *z* moves along the path and the effect of different choices of the parameter $d^{*}$, which determines the value at which *z* inverts direction.

### Transitions Between Classes

**Fig. 11:**
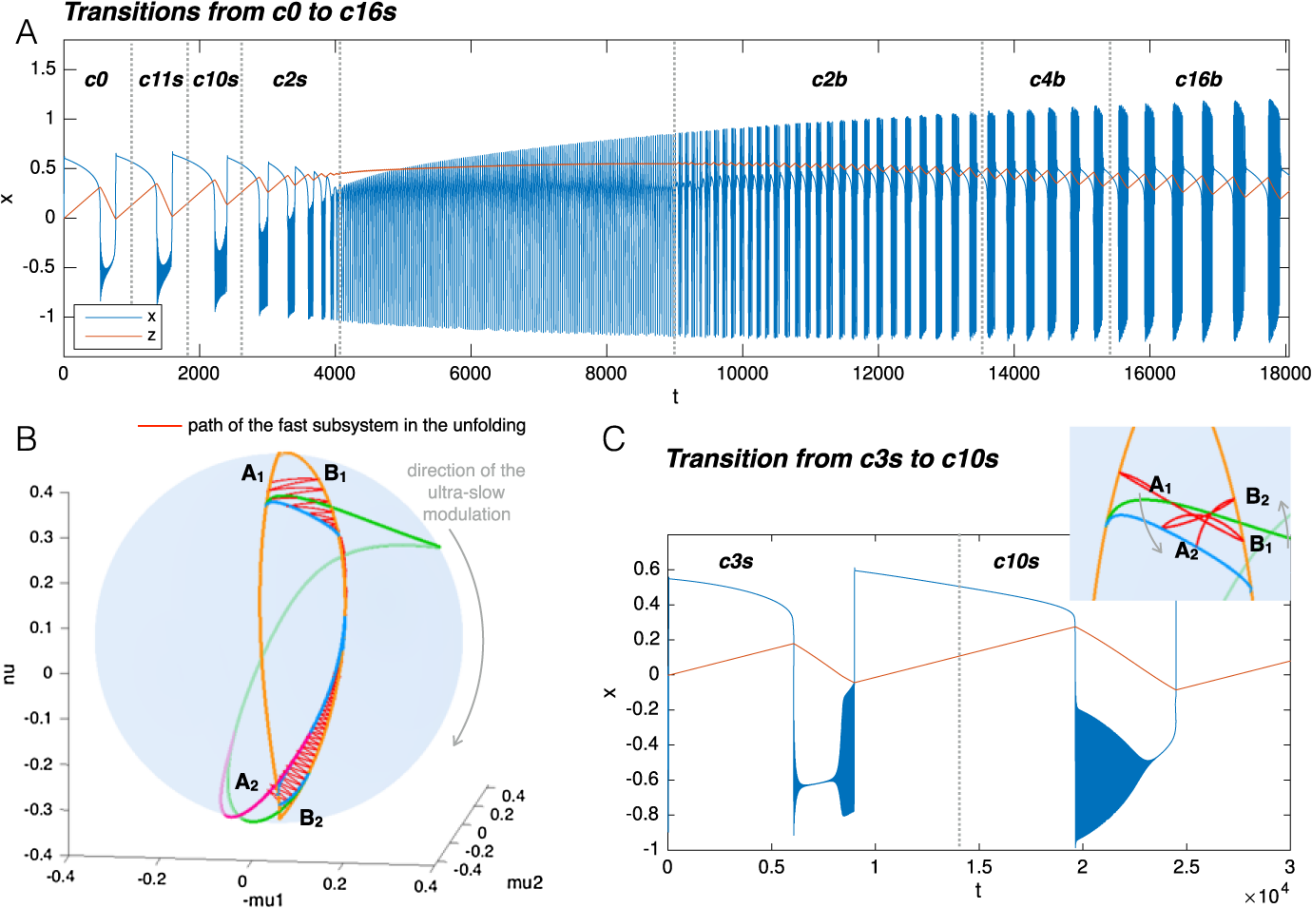
Transitions among classes. Transitions between bursting classes can be obtained through an ultra-slow modulation of the starting and ending points of the path followed by the fast subsystem in the unfolding. We here report two examples. **A** Simulated timeseries for the fast variable *x* and the slow one *z*. *Dotted vertical lines* mark the separation between different bursting classes. **B** The path followed in the unfolding is displayed in *red*. In the example portrayed, bursting activity moves from the LCs region (SN/SN, supH/supH, supH/SH, SN/SH) in the upper part of the unfolding, through a region with only oscillatory behavior, to the LCb region (SN/SH, SN/FLC, subH/FLC) in the lower part of the unfolding. The ultra-slow modulation pushes $\mathbf{A}_{1}$ and $\mathbf{B}_{1}$ downwards along arcs of great circles joining them with $\mathbf{A}_{2}$ and $\mathbf {B}_{2}$ respectively. **C** In this second example class SN/supH (c3s) switches to supH/SH (c10s). We show the timeseries and a zoom of the LCs region of the unfolding where the bursting takes place, with the path followed by the fast subsystem in *red*

The fact that the hysteresis-loop model produces bursting activity of one class rather than another only depends on the path followed in the unfolding, in our case, on the arc on the sphere. The settings that, in the present work, determine this arc are the two points **A** and **B**. Ultra-slow modulations of these points can determine a change of classes. Two examples are shown in Fig. 11. Figure 11A is the timeseries of a concatenation of transitions. The path followed by the system in the unfolding is shown in red in Fig. 11B. To obtain these transitions we have implemented a straight downward ultra-slow movement of the initial and final points of the path (the initial point **A** moves along the great circle linking $\mathbf{A}_{1}$ to $\mathbf{A}_{2}$, in the same way the final point **B** moves from $\mathbf{B}_{1}$ to $\mathbf{B}_{2}$). The system is initially in the LCs region in the upper part of the unfolding. The initial bursting class is c0, changes to c11s, c10s and c2s to end in a region with just oscillatory, not bursting, activity. Note that in this region, by construction, time-scale separation does not hold any more. The system then enters the LCb region in the bottom part of the unfolding starting with c2b, through c4b to c16b. There are two classes not present in this concatenation: c3s and c14s. It is not possible to obtain a sequence with both c10s and c3s, or with both c4b and c14s if the points **A** and **B** move along the arc with the same orientation, as in the simple slow modulation of the path we have implemented. More complex modulation could be used to obtain different transitions. In Fig. 11C we propose a transition between c3s and c10s, obtained by letting the point **A** drifting downward and the point **B** upward.

In general, transitions of classes are possible within the same region (LCs or LCb). To have a transition between classes in different regions, the system has to go through a simple oscillatory phase or a simple silent phase (as for example in the central part of Fig. 11A).

It is important to stress that **A** and **B** do not need to be, in general, the ending points of the path followed by the system, but determine the great circle it lies on, the direction of movement and the starting point. As *z* inverts its direction, those bifurcation points have the role of limiting the arc followed by the system. For this reason, even though in Fig. 11B we moved **A** and **B** downwards following the arcs connecting $\mathbf{A}_{1}$ to $\mathbf{A}_{2}$, and $\mathbf{B}_{1}$ to $\mathbf{B}_{2}$, respectively, the actual zig-zag path followed by the fast subsystem is determined by the bifurcations that close the hysteresis loop.

This is not true for slow-wave bursters, in which the whole trajectory in the unfolding must be specified. For this reason, and for the fact that path shapes are specific to each class, transitions between these kinds of bursters may be more difficult to implement.

### Slow-Wave Bursters

#### Slow-Wave Bursting Classes

**Fig. 12:**
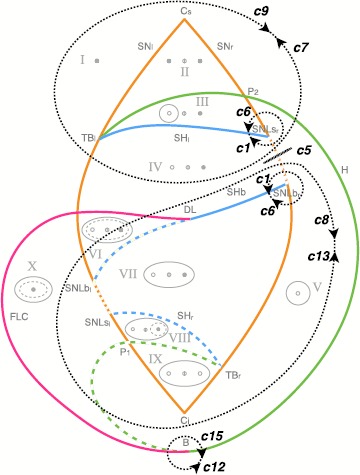
Paths for slow-wave bursters. We show examples of closed paths for the slow-wave bursting classes (*dotted black curves*). Paths for classes for which an hysteresis-loop counterpart exists are not shown, the hysteresis-loop path can, in fact, be used also for slow-wave bursting

Slow-wave classes do not require hysteresis and it is possible to use closed paths along which the system will move in a given direction [17]. We identified paths for all sixteen slow-wave bursting classes. Examples of these paths are shown as black dotted closed curves in Fig. 12. When paths for hysteresis-loop bursting exist, they can also be used to implement slow-wave bursters if the slow subsystem can oscillate back and forth the path without feedback from the fast one. For this reason, we show in Fig. 12 only the classes that do not have a hysteresis-loop counterpart in our model. Among these, c7, c8, c9 and c13 were not identified before in this unfolding.

#### Slow-Wave Bursting Model

Slow-wave bursting classes for which we have an hysteresis-loop bursting path in the model can be simulated using Eq. (6) if *z* is substituted with a two-dimensional self-oscillating slow subsystem, that is, $\mathbf{z}\in\mathbb{R}^{m}$, $m=2$. We did not produce these simulations. For the simulation of the other slow-wave classes, we substituted Eqs. (7) and (8) with an appropriate parametrizations of the closed paths shown in Fig. 12 (see Sect. 5.5) and set the slow dynamics to $\dot{z}=c$.

### Summary for the Codim-3 Deg. TB, Focus Case

**Fig. 13:**
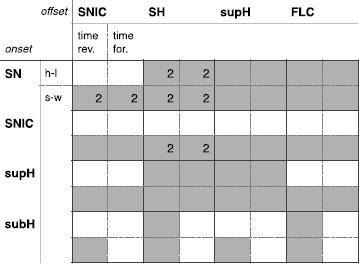
Focus bursters—summary. *The table* summarizes the results for the deg. TB singularity, focus case. *Rows* represent the four onset bifurcations, *columns* the four offset bifurcations. For each class we distinguish (*sub-columns*) the cases in which the equations of the unfolding are used with time forward or time reversed. With the sub-rows we distinguish the existence of hysteresis-loop (*h-l*) or slow-wave (*s-w*) classes. In *gray* we mark the existence of a given class. *The number 2* inside a cell indicates that for that class two realizations in different regions of the unfolding are possible. Overall the highest amount of classes is obtained in the time reversed case: all the sixteen slow-wave are in there and seven hysteresis-loop ones (one of them, c2, with two realizations)

Overall, in the unfolding of the codim-3 TB bifurcation of focus type, with time reversed, we found seven classes of hysteresis-loop bursters (one of them with two different realizations, one in LCs and the other in LCb) and all the sixteen classes of slow-wave bursters. These results are summarized in the table in Fig. 13. For each class we report whether a path exists in the time reversed or time forward condition (sub-columns) and whether the path is for hysteresis-loop (h-l) or slow-wave (s-w) bursting (sub-rows). Existence of the path is marked with a gray cell. In the table we report also results for a similar analysis conducted in the time forward condition (more details can be found in Sect. 5.6).

### Deg. TB Singularity—Elliptic, Saddle and Cusp Cases

The results described up to now pertain to the deg. TB focus case. We similarly investigated the unfoldings of the other three cases, the elliptic, saddle and cusp deg. TB singularities [22–24]. This did not add any new class with regard to those located in the focus case.

#### Elliptic

The elliptic case is described by Eq. (2) as well, but this time $b>2\sqrt{2}$. Baer et al. [24] showed that the description of this unfolding is topologically equivalent to that of the focus case. We thus have the same bursting classes as in the latter. The authors pointed out that in the elliptic case the small limit cycle displays a ‘canard-like’ behavior close to the SN curves, with rapid changes in the amplitude. This renders the numerical continuation of the limit cycle harder than in the focus case. Another difference underlined by Baer et al. is that in the elliptic case, the orbit at SHb tends to the boundary of the elliptic sector rather than to the origin when approaching the codim-3 singularity.

#### Saddle

**Fig. 14:**
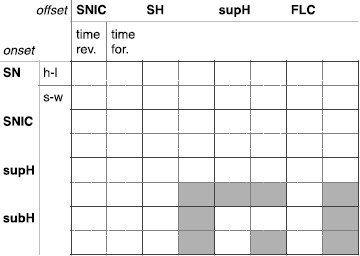
Deg. TB singularity: saddle and cusp cases. *The table* summarizes the results for the existence of bursting paths in the saddle case (same legend as in Fig. 13). The results for the cusp case are identical to those of the saddle case, where time forward and time reversed conditions are inverted, we thus do not report them in a extra table

The saddle case is obtained from Eq. (2) when the term $x^{3}$ has negative sign and for every *b*. In this case two of the three fixed points are saddles, which reduces the possibility of having bistability. We found, in fact, only two hysteresis-loop bursters and five slow-wave ones. Results are summarized in the table in Fig. 14. More details, including examples of bursting paths, can be found in Sects. 5.7 and 5.8.

#### Cusp

The equations for the cusp case are different from the equations for the other cases [22] (see Sect. 5.7). Unlike the other cases the cusp case only allows for two fixed points: one saddle and one focus. Bursting classes are the same as for the saddle case, but the time forward and time reversed behaviors are inverted. More details can be found in Sects. 5.7 and 5.8.

### Bursting Classes in the Partial Unfolding of the Doubly Degenerate TB Singularity

We have discussed in Sect. 2.5 the topology of the bifurcation diagram produced by the intersection of a sphere centered in the codim-3 singularity and the unfolding of this singularity. Bifurcation diagrams obtained for different values of the radius of the sphere are topologically equivalent provided the radius is small enough [23]. Krauskopf and Osinga [26] showed that for increasing values of *R* one can observe a sequence of different topologies for the bifurcation curves. The authors explain how changing *R* corresponds to changing the parameter *b* in Eq. (2) and to exploring the partial unfolding of the codim-4 doubly degenerate TB singularity. We numerically reconstructed the changes in topology identified in [26], with the goal of investigating how the paths for bursting activity are affected and whether new classes can be identified. It is important to stress that, while the paths for bursting discussed for small values of the radius (Sects. 3.1 and 3.4) can also be found in any arbitrary system exhibiting a deg. TB singularity, this is not necessarily true for the new paths we will describe in this section. These new paths, however, can be found in any system exhibiting a doubly degenerate TB singularity.

**Fig. 15:**
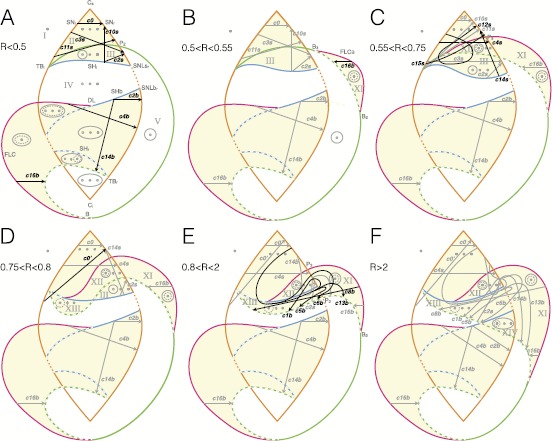
Effect of increasing the radius of the sphere. Bifurcation diagrams on spheres of different radius are topologically equivalent if $R<0.5$ (**A**). For bigger values of the radius, Eq. (2) does not describe the unfolding of the codim-3 deg. TB singularity anymore, but the partial unfolding of the codim-4 doubly degenerate TB singularity. Different topologies of bifurcation curves appear as shown in **B**–**E**. For each of these stages we verified the existence of bursting paths. Paths for classes already existing for smaller values of *R* are shown as *gray arrows*, paths for new classes as *black arrows*

In Fig. 15 we labeled different stages of topological equivalence with letters from A to F. The upper region of the bifurcation diagram is the most affected by changes in *R*. At first (B) a new curve of fold limit cycle bifurcation, FLC_*B*_, separates from the H curve in the part of the unfolding where only a fixed point exists. This curve later (C) crosses SN_*r*_ and enters the region where three fixed points coexist. The next topological changes are due to the behavior of the H bifurcation curve in the upper part of the diagram. This curve gradually passes below SH_*l*_ (D–E) until it intersects SHb (F). We evaluated the value of the radius for each stage with a precision of 0.05.

Bifurcation diagrams that correspond to layers of the unfolding of the deg. TB singularity have been identified in different models, for example the Bazykin predator-prey model [33, 36] or several neuron models (see Kirst et al. [31] and references therein). Other authors realized that the bifurcation diagrams identified in the models under investigation were deformations of this unfolding [37]. Studying the partial unfolding of the codim-4 doubly degenrate TB singularity can help to understand some of these deformations. For example, the bifurcation diagram for the Morris–Lecar model produced by Govaerts et al. [37], which appears also in a model for a compression system [38], can be located in the stage (E) in Fig. 15. The presence of the other stages in the right ordering would be indicative for a codim-4 doubly degenerate TB singularity in the model.

Do classes, which were found for small values of the radius, survive far from the codim-3 singularity? We examined each of the bifurcation diagrams in Fig. 15 looking for paths for bursting activity. We observed that some of the classes persist through all the values of *R* analyzed: they are all the classes in LCb (c2b, c16b, c4b, c14b), plus c0 and c2s in LCs. The other classes found for a small radius (in A) disappear after C.

**Fig. 16:**
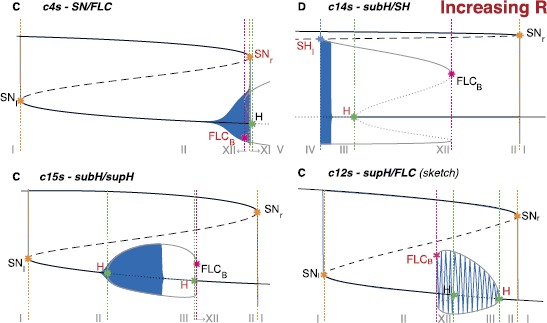
New classes in the codim-4 unfolding. Bifurcation diagrams for the new classes found when increasing *R* not involving SNIC onset and/or offset. *The capital letter* before the class name indicates to which stage, among those shown in Fig. 15 belongs the path used for the bifurcation diagram. For c4s (stage C) and c14s (stage D) we used arcs of great circles as paths, while for c15s (stage C) we used an arc of circumference different than the great circle. We could not find an arc as path for c12s (stage C), and the bifurcation diagram shown is a sketch, as well as the superimposed bursting trajectory. The latter is drawn with the purpose of clarifying where bursting occurs and is not meant to be realistic

**Fig. 17:**
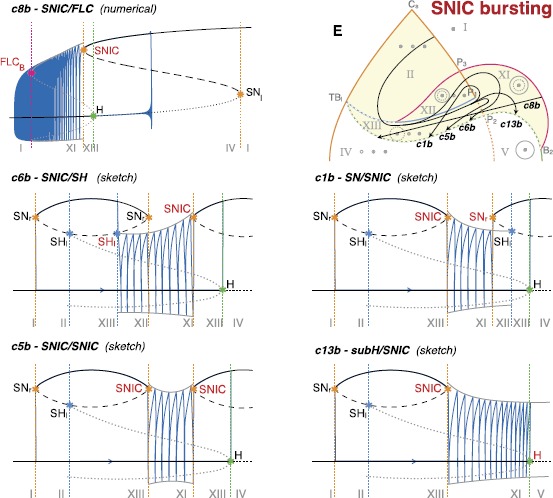
SNIC bursting. Bifurcation diagrams for the new classes found in the codim-4 unfolding, having SNIC onset and/or offset. *On the right in the first row*, we show a zoom of the region of interest of stage E where the paths can be found. The path for c8b could be parametrized as an arc of great circle, for this class we show the real bifurcation diagram and bursting trajectories. Paths for the other classes shown are more complex and we here propose sketches of the bifurcation diagrams and bursting trajectories. The bursting trajectory is drawn with the purpose of clarifying where bursting occurs and is not meant to be realistic

On the other hand, there are some classes that arise for bigger values of the radius, namely classes c4s, c8b, c14s, c13b, c12s, c15s, c5b, c1b and c6b. While two of them, c4 and c14, already appeared for $R<0.5$ with a different realization (i.e. c4b and c14b), the others were not found for a small radius and were not identified in the literature in the unfolding of the doubly degenerate TB singularity. Bifurcation diagrams for the new bursting classes not involving a SNIC bifurcation are shown in Fig. 16, those having a SNIC bifurcation at onset and/or offset in Fig. 17. In this codim-4 unfolding is also present another type of point-point burster, the saddle-node/subH pseudo-plateau burster identified by Osinga et al. [20], that appears at stage D and persists through stages E and F. We labeled it c0’.

We were able to find paths that could be parametrized as arcs of great circles for c4s, c14s and c8b (the only one with SNIC bifurcation). For class c15s this was not possible. To simulate this class and produce its bifurcation diagram we chose as a path the circumference crossing both the supercritical and the subcritical branches of H, together with the SN_*r*_ curve. This is not a great circle as it is not centered on the origin. To simulate this class, we modified Eq. (7) as described in Sect. 5.9. With regard to c8b, the only class with SNIC bifurcation here identified for which the path can be parametrized as an arc of great circle, it has an anomalous bifurcation diagram as compared to the other classes investigated up to now: in this case the lower branch of fixed points, and not the upper branch, plays the role of silent state and we had to modify Eqs. (7)–(8) and Eq. (6) accordingly (details can be found in Sect. 5.9). Bifurcation diagrams for c4s, c14s, c8b and c15s are obtained numerically together with the superimposed bursting trajectories.

For the other new classes (c14s, c12s, c1b, c5b, c6b and c13b) instead, paths are more complex than arcs of circumferences. We did not parametrize these paths, and the bifurcation diagrams in Figs. 16 and 17 are sketches. In all the classes with SNIC the role of silent state is played by the lower branch of the equilibrium manifold, as in c8b.

**Fig. 18:**
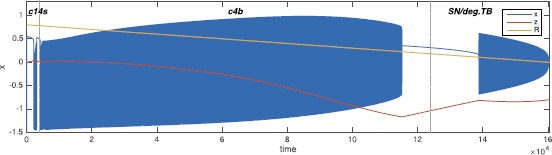
Transitions across spheres. This timeseries is an example of transitions among classes due to a linear ultra-slow modulation of the radius *R* (in *yellow*). The ${\theta, \phi}$ coordinates of **A** and **B** were kept constant and had the same values used to simulate c14s (at stage D). The next burst starts through SN_*r*_ and it ends (around $R=0.22$) through FLC. The third burst starts again on SN_*r*_ and the oscillations stop at $R=0$ when the fast subsystem undergoes the codim-3 deg. TB bifurcation

Transitions between classes are possible also by changing the value of the radius of the sphere, so that the bursting path is forced to cross spheres with different topologies of bifurcation curves. As an example, we implemented an ultra-slow modulation of the radius *R*, which decreases linearly from $R=0.8$ to zero (Fig. 18). The $({\theta,\phi})$ coordinates of **A** and **B** were kept constant and chosen so that the first burst is c14s (at stage D). The next burst starts through SN_*r*_ and then it ends (around $R=0.22$) through FLC. The third burst starts again on SN_*r*_ and the oscillations stop at $R=0$ when the fast subsystem undergoes the codim-3 deg. TB bifurcation.

## Discussion

### A Unifying Framework for Fast–Slow Bursters

Here we have shown how to build fast–slow planar bursters by appplying the unfolding theory approach to the unfolding of the codim-3 degenerate Takens–Bogdanov singularity [19]. We systematically explored the four possible unfoldings of this planar singularity: namely the focus, elliptic, saddle and cusp case [22–24]. For each of them we checked the time forward and time reversed behavior and located paths for slow-wave and hysteresis-loop bursters. We discovered that the focus case with time reversed covers the largest number of bursting classes. In fact, many bursting classes had already been identified in this unfolding by other authors [3, 19, 20, 25]. The other three cases did not bear new classes. In the unfolding of the focus case we found all the sixteen classes for slow-wave bursting and seven of the hysteresis-loop classes predicted as possible for planar bursting by Izhikevich [17]. We could identify seven additional hysteresis-loop classes when exploring the equations far from the codim-3 singularity, that is, the partial unfolding of the codim-4 doubly degenerate TB singularity [20, 26]. The labels used for the bursting classes can be found in Table 2.

**Fig. 19:**
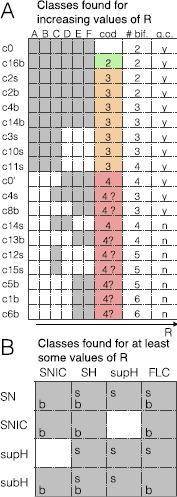
Existence and complexity of classes. **A** *The letters from A to F* refer to the stages of topological equivalence identified in Fig. 15 for increasing *R*. Some of the classes that were found for small *R* disappeared far from the codim-3 singularity, while new classes appeared when exploring the codim-4 unfolding. The figure shows the lifespan of each class as a function of *R* (*gray* indicates the existence of a class). For each class we report the smallest codimension of the local singularity in which unfolding the class first appears. This can be used as a measure of complexity. We identified hysteresis-loop bursters of complexity 2, 3 and 4 (*green*, *orange* and *red*). For the classes with a question mark, further investigation is needed to establish their complexity in terms of codimension. As an additional element to understand the complexity of a class, we report the minimum number of bifurcation curves that the path for that class here identified has to cross, which may also affect whether an arc of great circle can be used as a path (*last column*, ‘*y*’ (yes) if the great circle ‘*g.c.*’ can be used, ‘*n*’ (no) otherwise). **B** *The table* summarizes the hysteresis-loop bursting classes present in our model, considering also changes in *R*. *The letters ‘s’ and ‘b’* indicate whether the silent state is, respectively, outside or inside the limit cycle of the active phase

When increasing the radius of the system, while some additional classes can appear, others are destroyed. The lifespan of each class, as a function of *R* is reported in Fig. 19A. Figure 19B summarizes the hysteresis-loop classes found in the unfolding, considering those located far from the codim-3 singularity. Overall, fourteen classes out of sixteen are present, some with a realization both in the LCs and in the LCb regions. We could not find classes SNIC/supH (c7) and supH/SNIC (c9), as there is no value of *R* in the investigated range for which both SNIC and supercritical Hopf bifurcations are present in the bistability region.

We built a model that can produce bursting activity for all the hysteresis-loop classes found for which the path can be parametrized as an arc of great circle. These classes are all those found for small *R* (c2s, c3s, c10s, c11s, c2b, c4b, c14b and c16b) plus c4s and c8b. The model can be extended to c15s with some modifications. The equations of the fast subsystem of the model are those of the unfolding of the focus case of the deg. TB singularity. The unfolding parameters are parametrized in terms of one slow variable to follow the required path. The slow variable shows oscillations thanks to feedback from the fast subsystem. It can be observed from the phase flows in Fig. 4 that the fast subsystem is globally stable in each parameter configurations. This implies that, when the fast subsystem is in the bursting region, for all possible initial conditions the fast dynamics will converge to either the stable fixed point or stable limit cycle. The slow variable, on the other hand, is designed to ensure the alternation between these two states. This implies that all initial conditions will lead to the same bursting dynamics if the system is in the bursting region. The only parameters that determine the bursting class, if any, are the starting and ending points of the path. All the other parameters of the model can affect the shape of the orbit followed by the system without changing the class. For example, we showed that they can increase/decrease the number of oscillations in the active phase, alter their amplitude and frequency (within the constraints imposed by the bifurcations involved), or modify the silent/active phases lengths ratio.

This provides a unifying framework to investigate the underlying mechanisms of systems able to display different bursting behaviors. Furthermore, transitions between classes can easily be implemented through an ultra-slow modulation of initial and final points of the path [21].

We could make some predictions about which transitions are easier to obtain and which instead require one to cross regions of the unfolding where the system is not bursting. We also clarified why transitions are easier to obtain for hysteresis-loop bursters than for slow-wave ones.

Submanifolds of the three-parameter unfolding of the deg. TB singularity can be identified in several neuron models and this unfolding has been proposed as a key element to understand neural excitability [30–32, 37, 39]. Our results allow one to extend the understanding of the dynamical repertoire hosted in this unfolding, by giving a complete description of the planar bursting activity that can be obtained thanks to a coupling of the planar unfolding with a slower system. This allows, once the codim-3 deg. TB bifurcations of a model have been identified, to predict all possible planar bursting behaviors if the model is used as fast subsystem; whereas, if the system exhibits the codim-4 doubly degenerate TB singularity, we can predict only some of the possible planar bursting classes, based on the partial unfolding of this codim-4 singularity [20, 26].

### Finding New Paths for Bursting

Bertram et al. [3] searched for bursting paths in the two-parameter bifurcation diagram of the Chay–Cook model. This bifurcation diagram, as pointed out by the authors, is a layer of the unfolding of the deg. TB singularity, focus case. It can be obtained by keeping $\mu_{2}$ fixed at a negative value. Such a layer describes a bifurcation diagram that excludes some of the points that we find on the sphere: the two cusp points C_*s*_, C_*i*_ (they require $\mu _{2}=0$), and the Bautin point (requires a positive $\mu_{2}$). In this layer, Bertram et al. identified c2s, c2b, c4b and c16b with hysteresis, and c5 without hysteresis. De Vries [25] added the path for c3s with hysteresis. The complete unfolding on the sphere has been investigated by Osinga et al. [20]. The authors located paths for the bursters known to Bertram et al. and de Vries and they added the path for c10s. By exploring the unfolding far from the codim-3 singularity they also added a new class. This extra class is the point-point SN/subH burster. Izhikevich [17] and Golubitsky et al. [19] identified additional slow-wave classes close to two codim-2 bifurcations that exists also in the unfolding of the deg. TB singularity: the SNL point (c1, c6) and the Bautin point (c11, c12, c15). In this article we have provided a systematic framework of bursting activity, located paths for bursting in parameter space and identified novel paths for c4s, c8b, c11s, c14b, c14s, c12s, c15s, c1b, c5b, c6b and c13b for hysteresis-loop and all the missing classes for slow-wave (excluding those for which there is already an hysteresis-loop path, they are c7, c8, c9 and c13).

### Complexity of Bursting Classes

Golubitsky et al. [19] introduced a notion of *complexity* in the characterization of bursters. They defined the complexity of a bursting class as the codimension of the lowest codimension singularity in which unfolding that class can be found. Onset and offset bifurcations, in fact, are not enough to describe the sequence of bifurcations required by a bursting class. Other bifurcations may be required to obtain a class and even a greater number of them may be needed for the hysteresis-loop types. If more bifurcations are required for a class, then more parameters must be tuned to obtain a given sequence. This increases the complexity of the class. The number of parameters to be tuned is reflected in the codimension of the singularity in which unfolding the class first appears. Thus, Golubitsky et al. argued that the more complex a class is, the less likely it is to encounter that class, in empirical data or in models. They proposed to complement Izhikevich’s classification by providing information on this measure of complexity.

With regard to hysteresis-loop bursters, the class of smallest complexity is subH/FLC (c16), which exists close to the codim-2 Bautin bifurcation as outlined by Golubitsky et al. Class SN/SH (c2) lives close to the codim-2 Saddle-Node-Loop bifurcation [17], but this bifurcation is not local and the lowest codimension singularity for this class is the codim-3 deg. TB one [19]. Figure 19 reports the complexity of the classes identified in the present work. We classified the classes appearing far from the codim-3 singularity, thus in the partial unfolding of the doubly degenerate TB singularity, as codim-4 classes. With regard to these classes, it is an open question whether they could be located in the proximity of a codim-3 singularity other than the deg. TB. As pointed out by Osinga et al. [20], not all the unfoldings of codim-3 singularities are available. Nonetheless, the authors noted that the unfolding of the deg. TB is the only one to present both a codim-3 cusp point and a codim-2 TB bifurcation (at which Hopf and saddle-homoclinic bifurcations coincide). From this we can conclude that classes that require all these conditions, such as c14s, cannot be found in the unfolding of other codim-3 singularities. Thus, their complexity is four. Further work is required to determine the codimension of classes c8b, c15s, c13b, c12s, c15s, c5b, c1b and c6b.

An additional indicator of the complexity of a class is given by the number of bifurcations that its path has to cross to produce the desired bursting behavior and to close the hysteresis loop. Among the classes of codim-3, for example, paths for c2 need only to cross saddle-node and saddle-homoclinic bifurcation curves, while, on the other extreme, c11s requires one to go through four bifurcation curves. This implies that a more refined tuning of the parameters of the path is required for c11s to obey all constraints. For this reason, we added in Fig. 19 a column stating the minimum number of bifurcations encountered by the paths we identified. When the number of bifurcations to cross is high, it may become harder to cross them using a path as simple as an arc of great circle. In this work, this was not possible for all the classes that needed six or five bifurcations, and for some of those needing four bifurcations.

In the present work, we focused on point-cycle bursters, that is, bursters with a fixed point like silent phase and with a limit cycle for the active phase. We also provided two examples of a point-point burster, in which both phases are given by fixed points. Other possibilities exist that we did not address, as for example cycle-cycle bursters, in which the silent phase is characterized by small amplitude oscillations, requiring the coexistence of two stable limit cycles.

### Modeling Approaches

In the present work we have considered bursting that can be obtained in systems with two fast variables and one or two slow variables. However, more than two time scales can interact to produce more complex bursting patterns not possible with only two time scales [40]. The oscillatory activity of the fast subsystem itself can arise from the interplay of multiple time scales [39]. Franci et al. [21] applied the unfolding theory approach to a codim-3 singularity to build a three-variable model for bursting activity with three time scales, motivated by the spike-like oscillations of neuronal bursters. They used the unfolding of the codim-3 winged cusp singularity, which is described by a single variable *x*. The unfolding presents regions with only one fixed point and regions with coexistence of three fixed points. No limit cycle can live in one dimension. To generate oscillations Franci and coworkers expressed the unfolding parameters in terms of a second variable *y* acting on a slower time scale. The second variable receives feedback from the fast one and exploits the hysteresis present in the fast variable to create a limit cycle in the plane $( x,y )$ (relaxation oscillations). This second variable plays the same role as our *z* in class c0 in Fig. 4: no limit cycle is present in the fast subsystem but, thanks to hysteresis, one can be created in the $( x,y )$ plane (note that the spiraling towards the fixed point in c0 is due to the presence of the second fast variable and is thus absent in the limit cycle generated by Franci et al. in the $( x,y )$ plane). The oscillations created by this limit cycle have a spike-like shape. The authors introduced a third variable, *z*, acting on a slower time scale than *y*, that allows for the alternation between active and silent phases with a similar mechanism of that used here. By changing the parameters they provided a model for c2, c3, c4 and c16 hysteresis-loop bursters. They also showed an example of how an ultra-slow modulation can lead to transitions between classes.

Our approach, based upon the planar unfolding of the codim-3 deg. TB singularity, allowed us to identify a richer repertoire of bursting activity using the same number of variables. This is due to the fact that the planar unfolding of the codim-3 deg. TB is richer, in terms of limit cycles and bifurcations, than the one-dimensional unfolding of the codim-3 winged cusp coupled with a slow variable. While the extra classes found with this method may not play a role in neuronal bursting [21], they could be relevant for other types of bursting system, for which oscillations are not necessarily spike-like, for example epileptic seizures. Another possibility to implement time-scale separation is to introduce it within the planar system of the deg. TB singularity. The slow-fast TB singularity has already been studied in [41] and appears in what has been proposed as a minimal model for neural excitability (displaying also canard explosions) [39]. The bifurcation diagram of this model, in fact, resembles a layer of the unfolding of the deg. TB singularity (focus or elliptic) which passes through the origin. It would be interesting to explore what is added, in terms of bursting dynamics, by the introduction of this third time scale within the deg. TB singularity.

The unfolding theory approach proves to be a valuable tool to build bursters when time-scale separation holds. When this does not happen, then different phenomena can occur, including chaos [17].

In our study bursting behavior was engineered by describing slow changing parameters. However, this is not the only way of eliciting bursts. Both slow and fast interventions can provoke bursts. For parameter changes the changes are usually slow (as discussed throughout the paper). However, the state variables are sensitive to perturbations, for example, because of multi-stability. In this study, the limit cycle that encircles all fixed points is less sensitive to perturbations than the limit cycle that does not encircle all fixed points (see Fig. 5). The effect of perturbations is mostly reversible in the latter case, where in the former case the fast event needs to be coordinated (e.g. temporally) to reverse an effect of perturbations (see Spiegler et al., [42], for example Figs. 10–12). The existence of a ‘small’ limit cycle next to others or fixed points can be directly used to describe bursting behavior by fast events, for instance, in repetitive spiking sequences. Other dynamics associated with bursting behavior are quasi-periodicity, deterministic chaos and intermittency [43].

## Methods

### Reproducing the Unfolding

To reproduce the unfoldings of the deg. TB singularity focus case (Fig. 3C and Fig. 4), we used the Matlab-based software Matcont and CL_Matcont [44]. We described the parameter space with spherical coordinates, fixed the radius $R=0.4$ and explored the $(\theta,\phi)$ parameter space. We computed the equilibrium manifold for a fixed value of *θ*, identified the SN points and performed their two-parameter continuations. From the Takens–Bogdanov points located on the SN curves, we started the continuation of the H and SH curves. From the Bautin point on H we computed the FLC curve and from the last point of the FLC curve we started the continuation of the SHb curve.

### Investigation of the State Space

To produce the phase flows in Fig. 5, we choose a point $(\theta,\phi)$ in parameter space for each region of the unfolding labeled with a Roman numeral. We computed *x* and *y*-nullclines analytically, while the orbits where simulated with Matcont.

To investigate the amplitude and frequency of the stable limit cycle, we discretized the parameter space with 720 points for *ϕ* and 360 for *θ*. For each point we integrated the fast subsystem (using Matlab function ‘ode45’) using as initial conditions: a big value for $(x,y)$ ($(x,y)=(10,10)$) so that the system stabilizes on the limit cycle in regions V to X; a point close to the fixed point on the lower branch (we added $\epsilon=0.01$ to the *y* coordinate of the fixed point) to reach the limit cycle in region III. We simulated 3000 s, removed the first 500 s to avoid the transient behavior and used the last 2500 s to compute the amplitude and frequency. For the amplitude we took the difference between the maximum and minimum of the timeseries; for the frequency we used Hann window and then applied Discrete Fourier Transform.

To obtain the flat representation we applied the Lambert Equal Area Azimuthal Projection, the Matlab identifier for this projection is ‘equaazim’.

### Hysteresis-Loop Bursting Classes

To locate paths for hysteresis-loop bursting activity we first identified the regions of the unfolding where there is bistability between a fixed point and a limit cycle. Bistability is, in fact, a necessary condition to have hysteresis. These regions are shaded in yellow in Fig. 4 and are labeled LCs and LCb. We listed the bifurcations present in each region (including those at the border of the bistability region) and divided them depending on whether they could be used for bursting onset, offset or both. We then listed all the pairs of onset/offset bifurcations possible for that region. Once compiled the list we verified for each entry the existence of a path crossing the right sequence of bifurcations to obtain a given bursting class, including those necessary to close the hysteresis loop (when different from the onset or offset bifurcations, they where SN_*r*_ and SN_*l*_). At this stage we did not put constraints on the shape of the path. We focused on sketching bifurcation diagrams based on the knowledge of the state space configuration for each region of the unfolding, and verified their compatibility with the presence of a bursting trajectory. We identified paths for all the possible classes listed, which guarantees that the analysis is complete and no other class lives in this unfolding. This does not prevent paths different from those here identified to exist for a given class. Each path has to lie on the spherical surface, and be contained in the bistability regions. In addition to these constraints there are other class specific constraints, summarized in Table 3.

**Table 3:** **Bursting classes in the unfolding of the deg. TB singularity, focus case**

Class	Label	Bifurcations sequence	Point **A**	Point **B**
*LCs region*				
SN/SH	c2s	SH_*l*_, SN_*r*_	SH_*l*_	SN_*r*_ ∈ [P_2_, SNLs_*r*_]
SN/supH	c3s	SN_*l*_, H, SN_*r*_	SN_*l*_ ∈ [TB_*l*_, C_*s*_]	SN_*r*_ ∈ [P_2_, SNLs_*r*_]
supH/SH	c10s	SH_*l*_, H, SN_*r*_	SH_*l*_	SN_*r*_ ∈ [C_*s*_, P_2_]
supH/supH	c11s	SN_*l*_, H, H, SN_*r*_	SN_*l*_ ∈ [TB_*l*_, C_*s*_]	SN_*r*_ ∈ [C_*s*_, P_2_]
*LCb region*				
SN/SH	c2b	SHb, SN_*r*_^a^	SHb ∈ [DL, SNLb_*r*_]	SN_*r*_ ∈ [SNLb_*r*_,TBr]
SN/FLC	c4b	FLC, SN_*r*_^a^	FLC	SN_*r*_ ∈ [SNLb_*r*_, TBr]
subH/SH	c14b	SHb, H^a^	SHb ∈ [DL, SNLb_*r*_]	H ∈ [B, TB_*r*_]
subH/FLC	c16b	FLC, H^a^	FLC	H ∈ [B, TB_*r*_]

For each of the possible classes in the LCs or LCb region we state the requirements in terms of sequence of bifurcations the system has to go through and location of the initial and final points of the path.

^a^These classes can cross SHb ∈ [SNLb_*l*_, DL], SH_*r*_, SN_*l*_ and SNIC ∈ [SNLb_*l*_, SNLs_*l*_] between the offset and the onset point, without destroying the bursting trajectory.

**Fig. 20:**
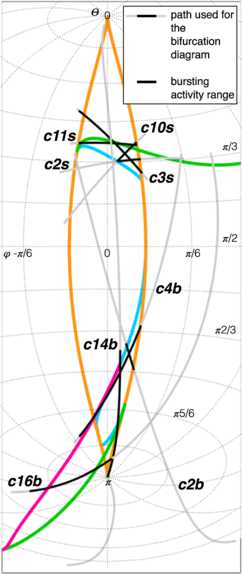
Real paths. This a flat projection of the unfolding of the focus case (same projection as in Fig. 9). We added the projection of the paths used for the bifurcation diagrams in Fig. 6 and the simulations in Fig. 8. *The black portion of the path* is where bursting takes place (i.e. where the slow variable oscillates). *The gray part* is the additional portion of the great circle explored to create the bifurcation diagrams in Fig. 6

At this point we verified that for each class it was possible to obtain a path with the desired properties by taking an arc of great circle, that is, the simplest possible path on the sphere. This was always possible and allowed us to use the same parametrization for all the classes of the model, with the initial and final points of the arc as free parameters. To verify the sketched bifurcation diagrams, we used these arcs parametrized in terms of *z* and, using Matcont, we produced the real bifurcation diagrams shown in Fig. 6. The arcs of great circles used for Fig. 6 are shown in Fig. 20 with the flat Lambert equal area azimuthal projection. In gray the whole range of the great circle used for the bifurcation diagrams in Fig. 6, in black the path on which the bursting trajectory is confined.

**Fig. 21:**
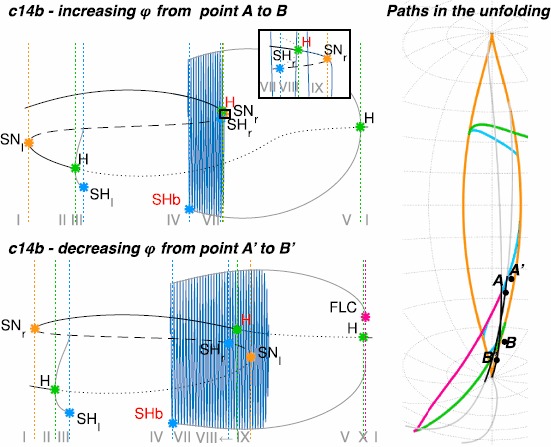
Different bifurcation diagrams for the same class. Different choices for the path for a class, within the constraints described in Sect. 5, can bring one to bursting of the same type but different bifurcation diagrams. Here, in particular, we present class subH/saddle-homoclinic (c14b) as an example. The example shows how having an increasing (*top bifurcation diagram*) or decreasing (*bottom bifurcation diagram*) *ϕ* when going from the offset point to the onset point can bring us to the same class, while the bifurcation diagrams show some differences. For example in the order in which SN_*r*_ and SN_*l*_ appear, or in the presence of FLC. *On the right* the flat projection of the paths used

The bifurcation diagram of a given class, even when using arcs of great circles as paths, may differ from those shown in Fig. 6 for the following reasons: there may be some variations in the sequence of bifurcations crossed within the bistability region LCb; different bifurcations may be encountered outside the bistability region depending on the great circle. As an example of the first situation we can consider the bifurcation diagram of c4b, which in Fig. 6 goes through regions I, IV, VI, VII, V, I, but would produce c4b bursting even with sequences such as I, X, VI, VII, V, I or I, X, VII, V, I or I, X, VIII, VII, V, I. An example of changes outside the bistability region is proposed in Fig. 21 for class c14b. In the figure we choose a path with increasing *ϕ* from **A** to **B** and a path with decreasing *ϕ*. Similar results can be shown for classes c2s, c10s, c2b, c4b and c16b, but are not reported here.

### Transitions

To obtain the transitions between classes in Fig. 11, we implemented an ultra-slow modulation of the **A** and **B** points. We considered four points on the unfolding: $\mathbf{A}_{1}$ and $\mathbf{A}_{2}$ for the initial point **A**, $\mathbf{B}_{1}$ and $\mathbf{B}_{2}$ for the final point **B**. We substituted the vectors **A** and **B** in the equations of the parametrization of the path, Eq. (7), with the following:

9 $$ \begin{aligned} \mathbf{A}&=\mathbf{A}(u)=R(\mathbf{g}\cos{u}+ \mathbf{h}\sin{u}), \\ \mathbf{B}&=\mathbf{B}(w)=R (\mathbf{l}\cos{w}+\mathbf{m}\sin{w}), \end{aligned} $$

where $\mathbf{g}=\mathbf{A}_{1}/R$ and $\mathbf{h}=((\mathbf {A}_{1}\times\mathbf{A}_{2})\times\mathbf{A}_{1})/\Vert(\mathbf {A}_{1}\times\mathbf{A}_{2})\times\mathbf{A}_{1}\Vert$ define the parametrization of the great circle from $\mathbf{A}_{1}$ to $\mathbf {A}_{2}$ in terms of *u*, and $\mathbf{l}=\mathbf{B}_{1}/R$ and $\mathbf {m}=((\mathbf{B}_{1}\times\mathbf{B}_{2})\times\mathbf{B}_{1})/\Vert (\mathbf{B}_{1}\times\mathbf{B}_{2})\times\mathbf{B}_{1}\Vert$ that of the great circle from $\mathbf{B}_{1}$ to $\mathbf{B}_{2}$ in terms of *w*. The dynamics of the ultra-slow variables has been chosen to be as simple as possible with:

10 $$ \begin{aligned} \dot{u}&=c_{A}, \\ \dot{w}&=c_{B}, \end{aligned} $$

with $c_{A}$ and $c_{B}$ constants. For Fig. 11A, B we used $c=0.002$, $c_{A}=0.0001$, $c_{B}=0.00012$, $d^{*}=0.3$, $\mathbf {A}_{1}=[0.2731,-0.05494,0.287]$, $\mathbf{B}_{1}=[0.243,0.0461,0.3144]$, $\mathbf {A}_{2}=[0.07337,-0.06485,-0.3878]$ and $\mathbf {B}_{2}=[-0.02792,-0.03676,-0.3973]$. For Fig. 11C $c=0.0001$, $c_{A}=0.00001$, $c_{B}=c_{A}$, $d^{*}=0.3$, $\mathbf {A}_{1}=[0.3454,0.02484, 0.2003]$, $\mathbf{B}_{1}=[0.29,0.06011,0.2689]$; $\mathbf{A}_{2}=[0.2731,-0.05494,0.287]$ and $\mathbf{B}_{2}=[0.3331,0.074,0.2087]$.

### Slow-Wave Bursting Classes

**Fig. 22:**
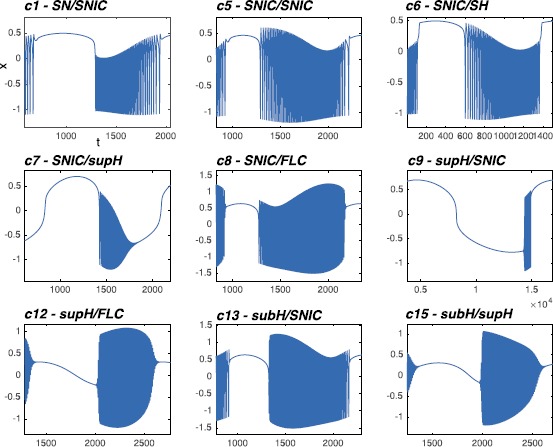
Timeseries of the slow-wave classes. Timeseries for slow-wave classes identified in the unfolding of the codim-3 deg. TB singularity. Paths used are circumferences defined by three points on the sphere, crossing the right sequence of bifurcation curves as sketched in Fig. 12

Paths for hysteresis-loop bursting can be used also for slow-wave bursting if the slow subsystem pushes the fast one back and forth along the path without the need of feedback from the latter. We searched the unfolding looking for paths for slow-wave bursting for classes of the taxonomy for which hysteresis-loop bursting was not possible and found all the classes of the taxonomy. Examples of these paths are shown in Fig. 12. We simulated these classes (Fig. 22) using as paths circumferences defined by three points on the sphere as done for c15s in Sect. 5.9, stage C. We modified the slow dynamics in Eqs. (4) and (6) using $\dot{z}=c$.

### Focus Case Time Forward Behavior

**Fig. 23:**
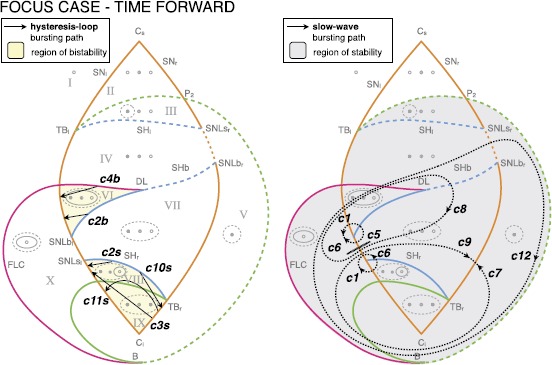
Focus case, time forward behavior. In *the left panel* we show hysteresis-loop bursting paths as *black arrows*. In *the right panel* slow-wave bursting paths as *dotted black closed curves*. *Arrows on the curve* indicate the direction of movement along the path

We considered the deg. TB bifurcation focus case, with time forward behavior [23], namely the system obtained by substituting $t'=-t$ in Eq. (2). The bifurcation diagram of the unfolding does not change but the stability of fixed points and limit cycles is affected, as shown in Fig. 23.

#### Hysteresis-Loop Bursting

There are two bistability regions: one in the central part of the bifurcation diagram and one in the lower part (in yellow in Fig. 23, left panel). In the first, the only bifurcation for the onset of the oscillations is the SN bifurcation, for the offset there are SH and FLC bifurcations. We identified paths for all the possible pairs: c2b and c4b. In this region the limit cycle surrounds the silent state (lower branch of the equilibrium manifold). In the lower part of the bifurcation diagram two possible onsets, SN and supH, and two possible offsets, SH and supH, give four possible pairs for which we located paths: c2s, c3s, c10s and c11s. The limit cycle surrounds the upper branch of the equilibrium manifold, while the role of silent state is played by the lower branch.

#### Slow-Wave Bursting

A requisite for bursting is that there should be at least one attractor at each point along the path. The area which satisfies this condition is colored in light gray in Fig. 23, right panel. The bifurcation curves within this area are: SN, SNIC and supH for onset; SN, SH, supH and FLC for offset. Bifurcations at the border of the region cannot be considered because there would be no attractor once the curve is crossed. We identified closed paths for all the possible pairs: 12 out of 16 classes are present (see Fig. 13). The four missing are those with subH.

### Saddle Case Time Forward and Cusp Case Time Reversed

Unfoldings for the saddle and cusp case are taken from Dumortier et al. [22, 23]. Equations for the saddle case can be obtained from Eq. (2), when the coefficient of the cubic term is negative. Equations for the unfolding of deg. TB singularity, cusp case are given by [22]

11 $$ \textstyle\begin{cases} \dot{x}={y,}\\ \dot{y}=x^{2}+\mu+y(\nu_{0}+\nu_{1}x\pm x^{3}). \end{cases} $$

**Fig. 24:**
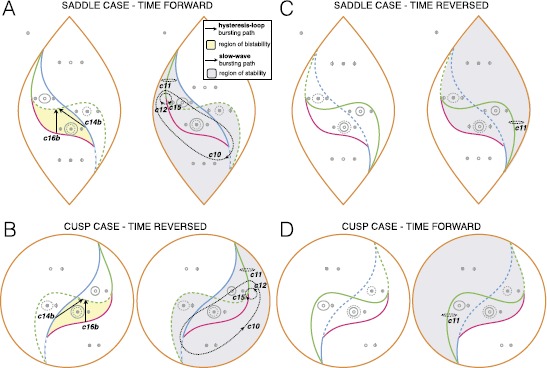
Saddle and cusp cases. Hysteresis-loop and slow-wave paths for **A** the saddle case, time forward; **B** the cusp case, time reversed; **C** the saddle case, time reversed, and **D** the cusp case, time forward. Existence of classes is the same for **A** and **B**, and for **C** and **D**

The saddle case with time forward behavior has the same bursting classes of the cusp case in the time reversed condition. The unfolding diagrams are shown in Fig. 24A, B.

#### Hysteresis-Loop Bursting

The bifurcation in the bistability region are: subH for onset; SH and FLC for offset. Paths for the two possible pairs, giving c14b and c16b bursting, can be located in the unfolding.

#### Slow-Wave Bursting

The bifurcation curves within the stability area are: supH and subH for onset; SH, supH and FLC for offset. Paths for all the possible pairs, for which an hysteresis loop has not been identified, can be found in the unfolding (c10, c11, c12 and c15).

### Saddle Case Time Reversed and Cusp Case Time Forward

The saddle case with time reversed has the same bursting classes of the cusp case in the time forward condition. The unfolding diagrams are shown in Fig. 24C, D.

#### Hysteresis-Loop Bursting

There is no bistability region, so hysteresis-loop bursting is not possible.

#### Slow-Wave Bursting

The only bifurcation within the stability region that can be used for seizure onset and/or offset is the supH. We located a path for supH/supH (c11) bursting.

### Hysteresis-Loop Bursting Classes in the Partial Unfolding of the Doubly Degenerate TB Singularity

To reproduce the bifurcation diagrams on spheres with different values of the radius described in [26], we proceed as in Sect. 5.1 with an additional step. We performed the numerical continuation of the FLC_*B*_ curve starting at another Bautin point identified through Matcont on the H curve. We repeated the procedure for different values of *R*, from $R=0.2$ to $R=3$ with step 0.05, to identify diagrams belonging to the different stages of topological equivalence labeled in Fig. 15 from A to F. We analyzed the bifurcation diagram at each stage looking for paths for bursting activity following the same procedure described in Sect. 5.3. We sketched bifurcation diagrams for each class based on the state space configuration for each region of the unfolding. For the new classes not present in stage A, we listed in Table 4 the requirements that a path must satisfy, besides being in the bistability region, regardless of its shape. We numerically verified the sketched bifurcation diagrams only for the new classes that were not present in the unfolding close to the codim-3 singularity. For some of these new classes we could not use an arc of great circle as a path, as specified in the detailed description below. For the classes found, which were already present at stage A, while the sketched bifurcation diagrams indicate that a path exists for that class, we did not verify that an arc of great circle could always be used.

**Table 4:** **Bursting classes far from the deg. TB singularity, focus case**

Class	Label	Bifurcations sequence	Point **A**	Point **B**
*LCs region*				
SN/FLC	c4s	SN_*l*_, FLC_*B*_, SN_*r*_; SN_*l*_, FLC_*B*_, subH, SN_*r*_	SN_*l*_ ∈ [TB_*l*_, C_*s*_]	SN_*r*_ ∈ [P_3_, SNLs_*r*_]
supH/FLC	c12s	SN_*l*_, FLC_*B*_, subH, supH, SN_*r*_	SN_*l*_ ∈ [TB_*l*_, C_*s*_]	SN_*r*_ ∈ [C_*s*_, P_3_]
subH/SH	c14s	SH_*l*_, subH, FLC_*B*_, SN_*r*_	SH_*l*_	SN_*r*_ ∈ [C_*s*_, P_3_]
subH/supH	c15s	SN_*l*_, supH, subH, FLC_*B*_, SN_*r*_	SN_*l*_ ∈ [TB_*l*_, C_*s*_]	SN_*r*_ ∈ [C_*s*_, P_3_]
SNIC/FLC	c8b	H, SNIC_*l*_, FLC_*B*_, SN_*r*_, FLC_*B*_, SH_*l*_, SNIC, FLC_*B*_; SN_*r*_, SH_*l*_, SNIC, FLC_*B*_	H ∈ [TB_*l*_, P_2_]	FLC_*B*_ ∈ [P_3_, B_2_]
SN/SNIC	c1b	SN_*r*_, SH_*l*_, SNIC, SN_*r*_, SH_*l*_, H, SN_*r*_, FLC, SH_*l*_, SNIC, SN_*r*_, SH_*l*_, H	SN_*r*_ ∈ [C_*s*_, P_3_]	H ∈ [TB_*l*_, P_2_]
SNIC/SNIC	c5b	SN_*r*_, SH_*l*_, SNIC, SNIC, H, SN_*r*_, FLC, SH_*l*_, SNIC, SNIC, H	SN_*r*_ ∈ [C_*s*_, P_3_]	H ∈ [TB_*l*_, P_2_]
SNIC/SH	c6b	SN_*r*_, SH_*l*_, SH_*l*_, SN_*r*_, SNIC, H	SN_*r*_ ∈ [C_*s*_, P_3_]	H ∈ [TB_*l*_, P_2_]
subH/SNIC	c13b	SN_*r*_, SH_*l*_, SNIC, H, SN_*r*_, FLC, SH_*l*_, SNIC, H,	SN_*r*_ ∈ [C_*s*_, P_3_]	H ∈ [P_2_, B_2_]

For each of the new classes found in the LCs region when increasing *R* we state the requirements in terms of sequence of bifurcations the system has to go through and location of initial and final points of the path. When more than one sequence is possible, they are all reported, but we did not verify that all the sequences could be obtained with paths as those described in the text for a given class.

The LCb region is not affected by changes in *R* (except in stage E; see below for details) and the classes leaving in that region persist through all the stages. Classes in the LCs region are, instead, influenced.

#### Stage B

A new separated region of bistability, XI, is created by the appearance of the FLC_*B*_ curve. The only onset bifurcation curve in the region is subH and the only possible offset bifurcation is FLC. We verified the existence of a sketched bifurcation diagram compatible with the existence of subH/FLC (c16b) bursting.

#### Stage C

The new region of bistability created by FLC_*B*_ merges with the LCs region (to distinguish it from LCb, we will continue to identify this region in the upper part of the unfolding with the label ‘LCs’, even though in the region XI the limit cycle surrounds the silent state). We now have in the bistability region three bifurcations for the onset (SN, supH and subH) and three for the offset (SH, supH and FLC). This leads to nine possible pairs (c2, c3, c4, c10, c11, c12, c14, c15, c16). We found paths for all of them and verified that the sketched bifurcation diagrams along these paths were consistent with the existence of bursting trajectories of the desired types. Some of these classes were not present in previous stages: c4s, c12s, c14s and c15s. We could use arcs of great circles as paths for c4s and c14s, while we used a different parametrization (see details below) for c15s. Bifurcation diagrams are shown in Fig. 16. Those for c4s, c14s and c15s are obtained numerically, that for c12s is a sketch.

For c15s, it was not possible for us to find an arc of great circle crossing in this order SN_*l*_, the supercritcal branch of H, the subcritical branch of H, FLC_*B*_ and SN_*r*_. To this goal we used an arc of a circumference on the sphere, different from a great circle, found by fixing three points on the sphere (rather than two points on the sphere and the center in the origin as for the great circle). The initial point **A** was lying on SN_*l*_ ∈ [C_*s*_, TB_*l*_], **B** inside region III and **D** on SN_*r*_ ∈ [C_*s*_, P_3_]. To parametrize the path we used the three points to compute the center **C** and the radius *r* of the circumference and defined the vectors $\mathbf{V_{AB}}=(\mathbf{A}-\mathbf{B})$, $\mathbf {V_{AD}}=(\mathbf{A}-\mathbf{D})$, $\mathbf{n}=(\mathbf{V_{AB}}-\mathbf {V_{AD}})/ \Vert\mathbf{V_{AB}}-\mathbf{V_{AD}}\Vert$, $\mathbf {e}=(\mathbf{A}-\mathbf{C})/\Vert(\mathbf{A}-\mathbf{C})\Vert$ and $\mathbf{f}=-\mathbf{e}\times\mathbf{n}$, so that the parametric equations of the circumference, starting in **A** and moving towards **B**, are

12 $$ \boldsymbol{\mu}(z)=\mathbf{C}+r (\mathbf{e}\sin{z}+\mathbf{f}\cos{z}). $$

For the bifurcation diagrams in Fig. 16 we used these values: for c4s $R=0.6$, $\mathbf{A}=[0.5225,-0.1454,0.2566]$, $\mathbf{B}=[0.5657,0.1638,0.1149]$, $c=0.00005$, $d^{*}=0.3$; for c14s $R=0.75$, $\mathbf{A}=[0.7148,0.2262,0.02091]$, $\mathbf {B}=[0.7117,0.2311,0.05016]$, $c=0.0000001$, $d^{*}=0.2$; and for c15s $R=0.6$, $\mathbf{A}=[0.4935,-0.1334,0.3142]$, $\mathbf {B}=[0.5775,0.0195,0.168]$, $\mathbf{D}=[0.5583,0.1606,0.1499]$, $c=0.00002$, $d^{*}=0.8$.

To obtain c12s we need to cross SN_*l*_, FLC_*B*_, the subcritical and the supercritical branches of H and SN_*r*_ in this order. This requires a path with a knot as shown in Fig. 15.

#### Stage D

At this stage, part of the H curve passes below SH_*l*_ and becomes entirely subcritical. This implies that all the classes present in the previous stage and having the supH bifurcation as onset (c10s, c11s, c12s) or offset (c3s, c11s, c15s) are not present anymore. There are not new bifurcations in the bistability region with regard to the previous stage, so new classes are not possible. We verified the existence of sketched bifurcation diagrams consistent with c2s, c4s, c10s and c16s bursting.

#### Stage E

The H curve is now completely below SH_*l*_ and part of the SNIC_*r*_ curve becomes part of the bistability region. We have three possible onset bifurcations (SN, subH and SNIC) and three possible offsets (SH, FLC and SNIC), which gives nine classes to verify. We found paths for all of them (c2s, c4s, c14b, c16b, c5b, c6b, c8b, c13b, c1b). All the classes not containing a SNIC bifurcation were already present in previous stages. For class c14b in LCs we could not represent the path with a straight line in the cartoon bifurcation diagram in Fig. 15 and we did not verify the required shape on the numerical bifurcation diagram. For this class it is the lower branch of the equilibrium manifold that plays the role of silent state. After the offset, at SH_*l*_, if the fast subsystem goes back to the silent state *z* changes direction and moves towards the onset. In this case the path will start on SH_*l*_, enter the XII region, then region XI to end on H. If the fast subsystem is attracted by the upper branch of the equilibrium manifold, *z* continues to decreases until SN_*r*_ is crossed and the fast subsystem settles in the silent state. In the latter case, the path followed will be longer and cross SN_*r*_, FLC_*B*_, SH_*l*_, SH_*l*_, SN_*r*_, subH as shown in Fig. 16.

Only one of the classes involving a SNIC bifurcation, SNIC/FLC (c8b) bursting, had a path that could be parametrized as an arc of great circle. In this class as well the role of the silent state is played by the lower branch of equilibrium. To simulate the timeseries we used the *x* coordinate of this lower fixed point for $x_{s}$ in Eq. (6). The values of the parameters used for the simulation are $R=1$, $\mathbf{A}=[0.8207,0.5572,-0.1263]$, $\mathbf {B}=[0.9455,0.3043,-0.116]$ and $c=0.001$. The numerical bifurcation diagram can be found in Fig. 16, the other bifurcation diagrams for SNIC bursting are sketches.

#### Stage F

The subcritical branch of the H curve now crosses also SHb and LCs merges with LCb. The bifurcations present in the bistability region are the same as in the previous stage, as well as the bursting classes.
